# Mesocosm Study of Chemical Treatments on Methane Emissions in Oil Sands Tailings Ponds – Part II: Illustrating the Relationship of Naphthenic Acids with Methanogenesis

**DOI:** 10.1021/acsomega.5c09941

**Published:** 2026-01-19

**Authors:** Xiaomeng Wang, Ian Vander Meulen, Dena W. McMartin, Chukwuemeka Ajaero, John Headley, Bipro Ranjan Dhar

**Affiliations:** 1 428248 Natural Resources Canada, CanmetENERGY Devon, 1 Oil Patch Drive, Devon, Alberta T9G 1A8, Canada; 2 Watershed Hydrology and Ecology Research Division, National Hydrology Research Center, Environment and Climate Change Canada, 11 Innovation Boulevard, Saskatoon, Saskatchewan S7N 3H5, Canada; 3 Department of Geography and Environment, 4512 University of Lethbridge, 4401 University Drive, Lethbridge, Alberta T1K 3M4, Canada; 4 Civil and Environmental Engineering Department, Faculty of Engineering, 3158 University of Alberta, 9211 116 Street, Edmonton, Alberta T6G 2H5, Canada

## Abstract

Naphthenic acid fraction compounds (NAFCs) are primary toxic components in oil sands process-affected water. Chemical treatments in tailings ponds can influence their concentration and composition, which may affect their toxicity. It is therefore crucial to measure the levels and distribution of NAFCs if there is any chemical treatment process being implemented in the tailings ponds. In the literature, some potential chemical treatment processes have shown promising results in terms of methane inhibition, thus reducing fugitive greenhouse gas (GHG) emissions from tailings ponds. However, little has been established to date on how the methanogenesis process along with chemicals used in the treatment affects the concentration and compositional characteristics of the NAFCs in the tailings ponds. In this study, laboratory scale bottle tests simulating oil sands tailings pond environments were used to investigate joint outcomes of chemical treatments on methanogenesis in relation to the concentrations and molecular level characteristics of NAFCs. Among the four chemicals (Na_2_MoO_4_·2H_2_O, Fe_2_(SO_4_)_3_, Na_2_SO_4_, and Na_3_C_6_H_5_O_7_·2H_2_O) tested, Fe_2_(SO_4_)_3_ reduced both methane production and the concentration of the NAFCs. There was also evidence for oxidative transformation of classical naphthenic acids (i.e., O_2_-NAFCs) presented in the study. These findings suggest the potential of using Fe_2_(SO_4_)_3_ as a chemical amendment for NAFC attenuation and methane reduction to assist with the cleanup effort for oil sands tailings ponds.

## Graphical abstract

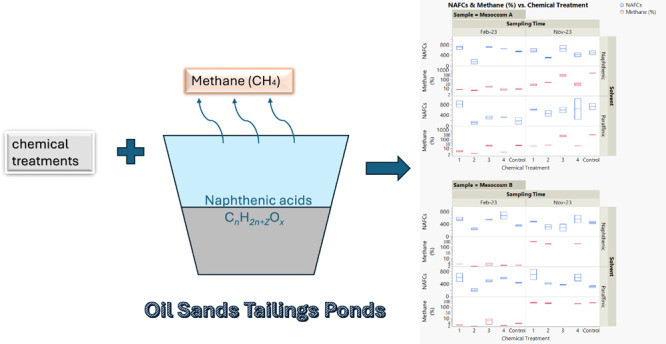

## Introduction

The oil sands of Alberta, Canada, constitute the world’s largest oil deposits, containing initial in-place reserves of 1.73 trillion barrels with an ultimate recovery potential of 314.5 billion barrels of bitumen.^1,2^ The three major oil sands deposits in the province of Alberta are located in Athabasca, Cold Lake, and Peace River, covering 142,200 km^2^ of land in the northern half of the province.^3^ Shallow oil sands deposits (i.e., up to 75 m) are accessed via surface mines, and bitumen extraction follows a modified version of the Clark Hot Water Process in which water is mixed with the ore and the bitumen is recovered from the floating froth formed in settling vessels.^4^ During extraction, organic acids (e.g., naphthenic acids) are leached from the oil sands into the water, named oil sands process water (OSPW), rendering it acutely and chronically toxic to aquatic organisms.^5^ Provincial environmental legislation prohibits the release of potentially toxic waste streams, thus the process water and waste fluid tailings containing these toxic chemicals are stored in on-lease oil sands tailings ponds.^4,6^ As of 2023, OSPW stored in tailings ponds reached a total volume of approximately 400 million m^3^ along with 1.5 billion m^3^ of fluid tailings.^7^

Due to their toxicity, there has been considerable research on naphthenic acids (NAs) in the past decade. Generally, concentrations of NAs in OSPW usually range from 40 mg/L to as high as 130 mg/L, whereas the natural concentration of these compounds in the Athabasca River and lakes in the region is typically less than 1 mg/L.^8,9^ Studies suggest that NAs can have a significant sublethal effect on aquatic life at concentrations of 15 mg/L.^10^ Besides the traditional classic NAs with a general formula of C_*n*_H_*2n*+*Z*_O_2_ (where *n* is the carbon number and *Z* represents the hydrogen deficiency), there are other acid extractable compounds in OSPW containing O_*x*_ (where *x* includes but is not limited to 2), nitrogen, sulfur, and aromatic structural moieties.^11–15^ Collectively, these acid extractable fractions are named naphthenic acid fraction compounds (NAFCs). The broader class of NAFCs including compounds containing O_3_, O_4_, and O_5_ may also contribute to the toxicity of OSPW.^16^

Another noteworthy problem in the oil sands industry is the degradation of air quality from greenhouse gas emissions. For instance, methane (CH_4_) is released from tailings ponds due to methanogenesis.^17^ Emissions of CH_4_ from tailings ponds can account for as much as 45% of the total CH_4_ emissions from oil sands facilities.^18^ Past work has sought potential methods or chemicals to reduce or inhibit methane emissions from oil sands tailings ponds.^19,20^ However, research to date has not jointly examined outcomes related to methanogenesis and concentrations of NAFCs, nor the impact of the treatment chemicals to the concentration and distribution of the naphthenic acids. As naphthenic acids are primary toxicants in tailings ponds,^21^ changes in their concentration and compositional characteristics will affect the toxicity of stored water. Thus, it is crucial to jointly measure how treatment such as chemical amendments affects both methanogenesis and the quantity and characteristics of NAFCs in tailings ponds. Although some naphthenic acids are recalcitrant chemicals^22,23^ and are not supposed to be readily available food sources for methanogens, these naphthenic acids can still degrade relatively slowly under anaerobic conditions.^24^ For instance, small fatty acids serve as substrates for methanogens.^25^ Since bioremediation of organic compounds in tailings pond is an attractive treatment option due to low cost and minimal waste generation,^26^ studies exploring the relationships of NAFCs with methanogenesis in oil sands tailings ponds may help identify candidate microorganisms or chemicals capable of significantly degrading NAFCs and other organics, empowering effective augmentation of tailings ponds or reclamation environments.^24^

In addition, the concurrent reduction of naphthenic acid fraction compounds (NAFCs) and methane emissions has far-reaching ecological implications in aquatic and broader environmental contexts. Lowering NAFC concentrations in process-affected waters alleviates acute and chronic toxicity to aquatic organisms, enhances microbial and algal community recovery, and improves the overall ecological resilience of receiving environments.^5,6,23^ Similarly, mitigating methane emissions from tailings ponds and anoxic sediments reduces greenhouse gas intensity while improving redox conditions and dissolved oxygen balance, thereby supporting more stable and biodiverse benthic habitats.^19,20^ Together, these measures contribute to the restoration of nutrient cycling, decrease sediment disturbance from gas ebullition, and minimize secondary contamination risks. In a broader environmental sense, coordinated reduction of both NAFCs and methane aligns local reclamation efforts with global sustainability goals by addressing the intertwined issues of aquatic health, carbon management, and climate change mitigation. However, ongoing monitoring is essential to assess potential transformation products of NAFC degradation and ensure that biogeochemical equilibria are maintained following emission reductions.

In this study, bottle tests using either simulated oil sands process water enriched with a mixed culture of methanogens or oil sands tailings with indigenous methanogens were incubated under laboratory conditions and monitored for methane production. The relationship of NAFCs with the methanogenesis process and chemical treatments simulating oil sands tailings pond environments has been investigated. The main goal of this work is to explore whether methane reduction by chemical treatment will affect the concentration and distribution of naphthenic acids. Ultimately, we would like to address both air and aquatic contaminants arising from oil sands tailings ponds by utilizing a chemical treatment that can lead to concurrent methane greenhouse gas reduction and attenuation of NAFCs.

## Experimental Section

### Bottle Setup

The detailed experimental procedure was provided in Part I of the two-article series.^27^ Briefly, two types of fluid fine tailings (FFT) sampled from the oil sands region in northern Alberta were used in this study, labeled as Mesocosm A samples and Mesocosm B samples. In addition, Mesocosm A samples were enriched with a mixed culture of methanogens, while Mesocosm B samples were made by indigenous methanogens from oil sands tailings. Paraffinic and naphthenic solvents were prepared in the laboratory by mixing representative solvents and used as readily available carbon sources to promote methane production in the tailings. Three chemicals (Na_2_MoO_4_·2H_2_O, Fe_2_(SO_4_)_3_, and Na_2_SO_4_) were used as potential methanogenic inhibitors, and another chemical (Na_3_C_6_H_5_O_7_·2H_2_O) was used as a potential methanogenic promotor for tailings. Due to the limited sample quantity, experiments were conducted in 100 mL mesocosm bottles for Mesocosm A samples (containing 70 mL of slurry in total) and 500 mL bottles for Mesocosm B samples (containing 333 mL of slurry in total) under anaerobic conditions.

In total, 20 bottles of Mesocosm A and 20 bottles of Mesocosm B were prepared, where 10 bottles of each were received premade naphthenic solvents or paraffinic solvents, respectively.^27^ This allowed for the preparation of each treatment condition (e.g., treatment/solvent pairing) in duplicate. The mesocosms were incubated in the dark at room temperature, and CH_4_ content in headspace was monitored biweekly. As described in Part 1 of the two-article series,^27^ 100 μL of headspace gas sample was taken from the bottles and injected into a gas chromatograph for methane analysis.

### Chemical Treatment

After establishing steady methane production from the bottles, four chemicals were added into the bottles at predetermined concentrations^28–31^ (shown in Table 1), each in duplicate. After chemical addition, these bottles were homogenized by a shaker for 30 min. Duplicate baseline controls (unamended mesocosms) were also prepared to monitor CH_4_ production without the addition of any methane suppressants or stimulants. Samples of the FFT slurry were taken periodically from the mesocosms for chemical analyses and microbial community characterization. The detailed results of the chemical analyses and microbial community characterization can be found in Part I of the two-article series.^27^ All of the experimental bottles were flushed with nitrogen after each sampling. The sampling frequency and schedule of events for the bottle test are shown in Figure 1.

**Table 1:** Chemical Addition to the Bottles

	Mesocosm A		Mesocosm B	
chemicals	concentration (mM)	concentration (ppm)	concentration (mM)	concentration (ppm)
Na_2_MoO_4_·2H_2_O	37.14	8986.71	20.00	4839.00
Fe_2_(SO_4_)_3_	32.19	12872.33	17.33	6931.25
Na_3_C_6_H_5_O_7_·2H_2_O	37.14	10923.71	20.00	5882.00
Na_2_SO_4_	96.57	13713.15	52.00	7384.00

**Figure 1:**
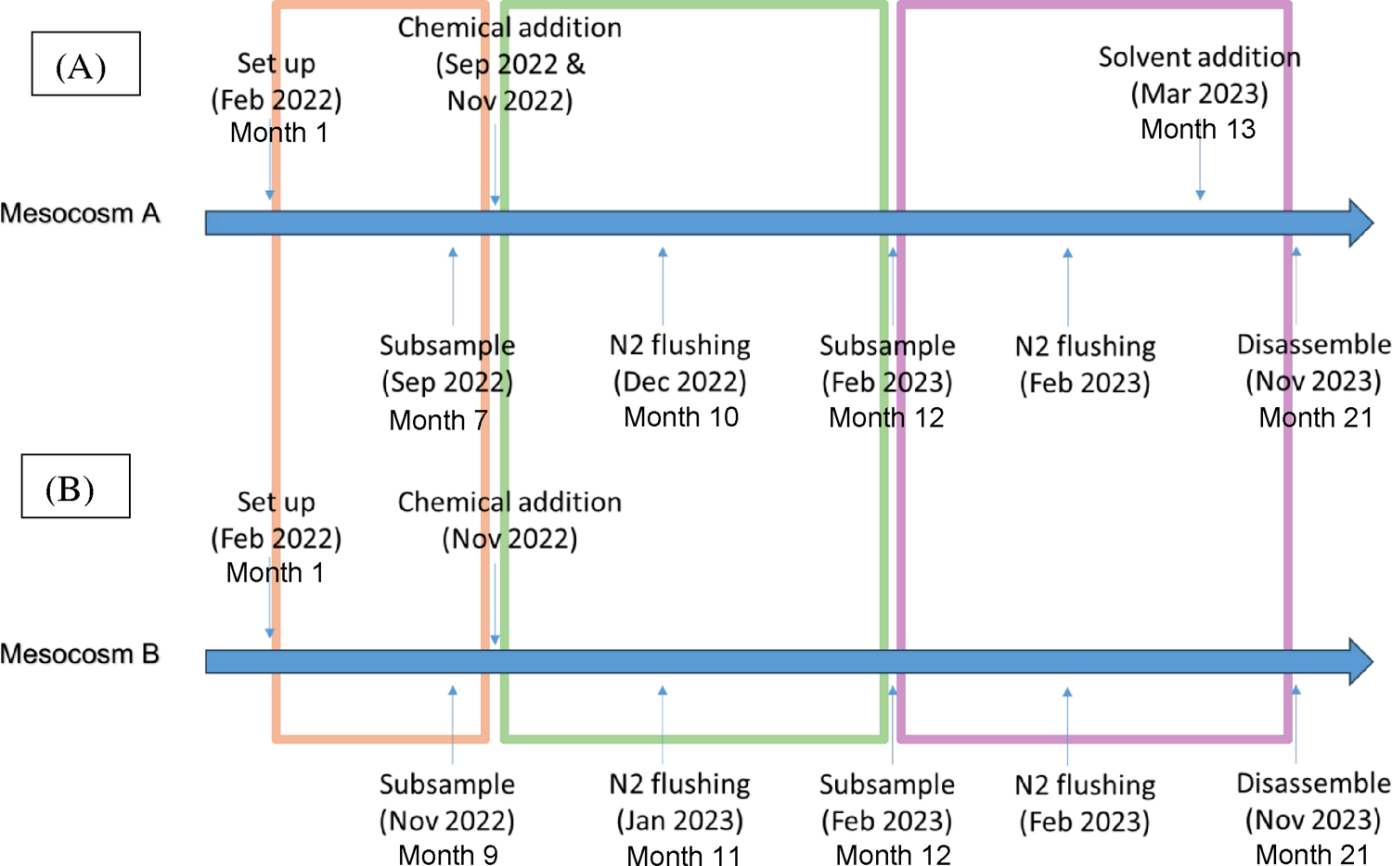
Sampling events during bottle test monitoring; (A) Mesocosm A bottles, (B) Mesocosm B bottles. Different phases of the sample monitoring are shown in different colored frames (orange: phase 1; green: phase 2 and purple: phase 3).

### Naphthenic Acid (NA) Analysis

Due to the limited water volume in the mesocosm bottles, tailings samples from the mesocosm were collected for NAFC analysis. Extractions were carried out where 15 mL of methanol was added to 5.0 g of wet solid material. The mixture was sonicated for 30 min and then centrifuged for 10 min at 5000 rpm. 7.5 mL of the supernatant was withdrawn from the sample into a separate clean test tube and evaporated under gentle N_2_ (5.0-grade; Linde Canada, Saskatoon, Saskatchewan) flow at approximately 40 °C to dryness. The sample was then redissolved into 1 mL of acetonitrile/Milli-Q water (1:1) containing 0.1% ammonium hydroxide solution. These reconstituted extracts were diluted in water to 100 mL, then treated as normal liquid samples, and subjected to a previously described SPE extraction^32^ for further isolation of analytes from fine solids and salts. Extraction cartridges containing 200 mg of ENV+ sorbent (Biotage, Charlotte, NC, USA) were rinsed with 1 column volume (6 mL) each of Milli-Q water, methanol, and further Milli-Q water to condition. Following column conditioning, sorbents were kept wetted, and samples were acidified to pH < 2 using excess formic acid (∼2 mL; ≥98%; Fisher Scientific, Oakville, ON, Canada). Acidified samples were then drawn through conditioned cartridges at 3–5 mL/min under gentle vacuum (approximately −50 to −100 mTorr). Loaded cartridges were then rinsed with a further column volume of Milli-Q water to remove residual solvents, and then a vacuum was drawn through cartridges under an ambient atmosphere until the sorbent was completely dry. Analytes were eluted from dry cartridges into clean, labeled glass culture tubes using 6 mL of methanol. Sample extracts were evaporated under gentle N_2_ (5.0-grade; Linde Canada, Saskatoon, Saskatchewan) flow at approximately 40 °C to dryness, then reconstituted in 1 mL of 1:1 acetonitrile/Milli-Q water (1:1) containing 0.1% ammonium hydroxide, vortexed for 10 to 15 s, and transferred to clean, labeled 2 mL amber glass LC/MS autosampler vials. All solvents used were HPLC grade (Fisher Scientific, Ottawa, ON, Canada).

Orbitrap MS analysis was performed using an LTQ Orbitrap Elite mass spectrometer (Thermo Fisher Scientific, San Jose, CA) operating in full scan in negative ion mode. Mass resolution was set to 240,000 (as measured at *m*/*z* 400) with an *m*/*z* scan range of 100–600. The ESI source was operated as follows: sheath gas flow rate of 10 (arbitrary units), spray voltage of 2.90 kV, auxiliary gas flow rate of 5 (arbitrary units), S lens RF level of 67%, heater temperature of 50 °C, and capillary temperature of 275 °C. The mobile phase used was 50:50 acetonitrile:water with 0.1% NH_4_OH. A flow rate of 200 μL/min was delivered by an Accela 1250 solvent pump (Thermo Fisher Scientific, San Jose, CA). A sample extract volume of 5 μL was injected into the mobile phase stream by using a Thermo PAL-HTC Accela autosampler (Thermo Fisher Scientific, San Jose, CA). The software used for instrument control/data acquisition and molecular analysis was Xcalibur version 2.1 (Thermo Fisher Scientific, San Jose, CA), and Composer version 1.5.2 (Sierra Analytics, Inc., Modesto, CA) was used to assign formulas to raw spectral data with an acceptable mass error tolerance of ≤3 ppm using C, H, O, N, and S to populate formulas. Acquired mass spectra were reproducible throughout the study (RSD ≤ 0.7%) and at a minimum, included duplicate analysis (*n* = 12).

Principal component analysis (PCA) was prepared in R 4.4.1 ^33^ by internally standardizing relative responses of all assigned formulas to base peak abundances and then arranging a standard matrix for subsequent PCA. Data were then normalized by Pareto normalization, which has been previously suggested as best practice for chemical data.^34^ PCA analysis was then carried out using the prcomp­() function, a default feature in R,^33^ which carries out PCA using a singular value decomposition technique. Visualizations of spectral data were all prepared using the ggplot2 function in R.^35^

### Dean–Stark Analysis

The concentrations of bitumen, mineral solids, and water of the tailings samples in the Mesocosm B after different chemical treatments were quantitatively determined by Dean–Stark analysis,^36,37^ where bitumen is extracted from the sample by heating toluene using the modified Soxhlet extractor apparatus. During the extraction process, the solids remain in the thimble; the water is collected in the water trap, and the bitumen is collected in the boiling flask, together with the toluene. At the end of the extraction, the weights of bitumen, solids, and water in the sample were acquired by using an analytical balance.

### Microtox Analysis

A BioTox WaterTox Standard Kit (*Aliivibrio fischeri* Toxicity Tests, formally *Vibrio fischeri*, Environmental Bio-Detection Products Inc., Burlington, Ontario, Canada) was used to assess toxicity of the samples according to the manufacturer’s protocol. The 81.9% Basic Test protocol was followed by 5 and 15 min incubation in the Microtox 500 Analyzer (Azur Environmental time). Samples were centrifuged for 1 h at 3600 rpm, then the supernatant was collected, pH was adjusted within the range of 6–8, and the solution was subsequently filtered through a 0.45 μm membrane filter and stored at 4 °C until analysis. Percentage inhibition was calculated after 5 and 15 min of exposure. It should be noted that 15 min of exposure is typically reported in the literature.^38^ A phenol standard (100 ppm) was used as a positive control to check the sensitivity of the luminescent bacteria before the analysis.

## Results and Discussion

### Headspace Methane Production Following Different Chemical Treatments in Mesocosms Amended by Solvents

The headspace methane monitoring is reported in part I of this two-article publication series.^27^ Briefly, we divided the entire methane monitoring period for the bottle samples into 3 phases (as shown in Figure 1). The initial period of methane production was categorized as phase 1, and steady methane production was observed by the end of phase 1. At the beginning of phase 2 during steady methane production, chemical treatments were applied to different bottles. By the end of phase 2, the tailings in the mesocosms were subsampled for NA analysis to investigate the impact of the chemical treatment on the compositional characteristics of NAFCs in the tailings solids. Phase 3 of the bottle monitoring began after the phase 2 subsample and lasted for about 9 months, and the goal of monitoring in this phase was to evaluate the long-term effects of the chemical treatments on the mesocosms.

In this paper, the analysis of variance (ANOVA) test was performed on headspace methane data to evaluate for statistical differences. For phase 2 of methane monitoring data, the highest methane production on one single date were used for statistical analysis. For phase 3 data, the averaged methane production over the entire period was used for statistical analysis. Under typical circumstances, the average methane production over the full experimental period would be the most appropriate metric for statistical analysis. However, in this study, the mesocosm designs differed in ways that prevented direct averaging of the Phase 2 data. Mesocosm A rapidly produced a large amount of methane in the early phase of the experiment and thus underwent multiple subsampling events and repeated N_2_ flushing during Phase 2, which periodically disrupted methane production. In contrast, Mesocosm B experienced uninterrupted gradual methane generation; thus, sampling occurred only at the end of Phase 2. Because these procedural differences artificially suppressed methane measurements in Mesocosm A, averaging Phase 2 data would produce misleading and noncomparable results between the two mesocosms. For this reason, the highest methane production observed on a single date during Phase 2 was used for statistical comparison.

As shown in Figure 2, compared to control samples (without chemical addition) in phase 2, chemicals such as Fe_2_(SO_4_)_3_, molybdate, and Na_2_SO_4_ significantly decreased methane production (*p* < 0.05). Sodium citrate slightly increased methane production, but the difference was not statistically significant (*p* > 0.5). A similar result was obtained when phase 3 methane gas production data were used for the analysis. The results are mostly aligned with expected outcomes.^19,39–41^ For instance, it has been reported that CH_4_ may not be detected in tailings samples until sulfate concentrations dropped to around 20 mg/L.^40^ Molybdate inhibited both sulfate reducing bacteria and methanogens, indicating a positive relation between the two processes.^39^ Trisodium citrate is an easily fermentable methanogenic substrate that may also contribute to tailings pond emissions.^42^ The only deviation we observed is in regards to the use of amorphous iron­(III) sulfate, as it has been reported that supplementing tailings with amorphous iron suppresses methanogenesis, whereas the crystalline mineral iron may enhance methane production.^19^ In comparison, our results show that Fe_2_(SO_4_)_3_ suppressed methanogenesis, despite being a crystalline iron mineral. Therefore, the detailed mechanisms of how treatment chemicals affect methane production warrant further study.

**Figure 2:**
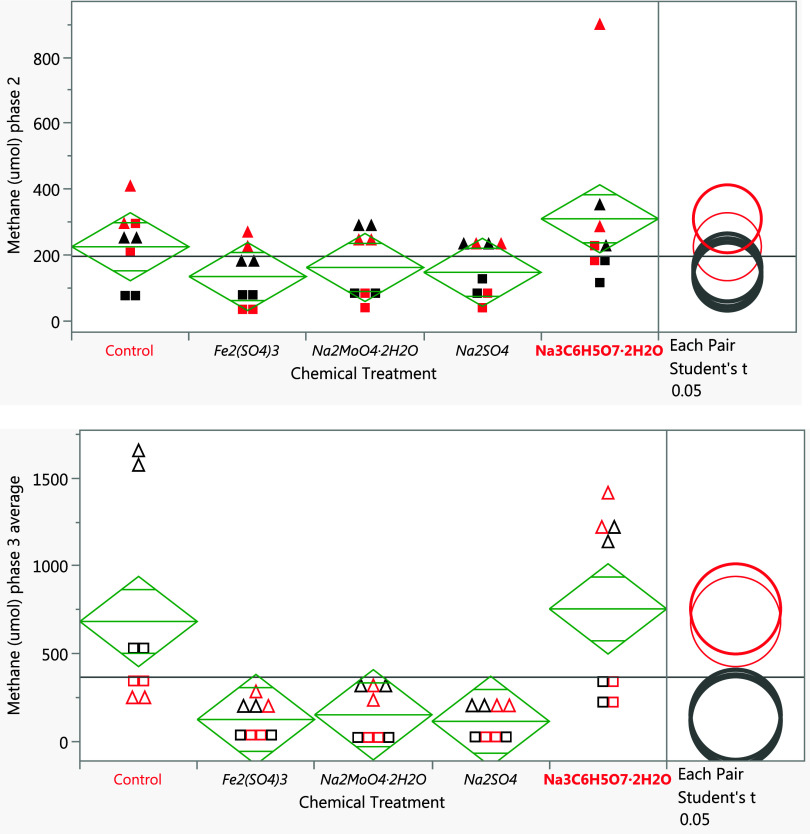
Analysis of variance (ANOVA) test for methane production in the headspace of the bottle samples after chemical treatments; phase 2 (top chart) Mesocosm A samples are in solid square and Mesocosm B samples are in solid triangle; phase 3 (bottom chart) Mesocosm A samples are in open squares and Mesocosm B samples are in open triangles. Samples spiked with paraffinic solvents are shown in red, and those spiked with naphthenic solvents are in black.

In particular, to investigate the impact of solvents and the source of tailings on the treatment effects of different chemicals, statistical analysis was performed on phase 2 methane production data isolating these factors. As demonstrated in Figure S1, the general trends of the treatment effects still hold: Fe_2_(SO_4_)_3_, molybdate, and Na_2_SO_4_ inhibited methane production, whereas sodium citrate stimulated methane production. However, it appears that inhibition was not very effective with Mesocosm B in phase 2, likely due to delayed methane production in this mesocosm.

In phase 3, Fe_2_(SO_4_)_3_, molybdate, and Na_2_SO_4_ inhibited methane production from Mesocosm A samples no matter which solvents were applied in the tailings (as shown in Figure S2). However, sodium citrate did not stimulate methane production in any of the mesocosm bottles, compared to the control samples. The reason may be due to the rapid consumption of sodium citrate in phase 2, which results in a lack of chemicals to stimulate methane production processes in phase 3. Similarly, Mesocosm B naphthenic tailings resulted in the same outcomes as compared to Mesocosm A samples (Figure S2), where Fe_2_(SO_4_)_3_, molybdate, and Na_2_SO_4_ significantly decreased the amount of methane production, but sodium citrate barely had any impact to the methane production. However, for paraffinic Mesocosm B, the amount of methane production from control samples was similar to the treated samples following chemical inhibition and citrate increased methane production. This could be due to the toxicity effects of the high solvent dosage in the Mesocosm B tailings and slow adaption to paraffinic solvents in the naphthenic solvent environments.^43^ Unfortunately, this makes the effects of chemical inhibitions uncertain, as the treatment effects were unclear in Mesocosm B paraffinic tailings. A longer monitoring period may be needed for future studies of these Mesocosm B samples.

The chemical treatments evaluated in this study seem to act directly on the microbial pathways responsible for methane production, offering a more rapid and controllable intervention. In contrast, biological strategies such as bioremediation, bioaugmentation, and phytoremediation operate on fundamentally different time scales and require strict environmental conditions, such as oxygen availability, stable vegetation, or long-term microbial acclimation. Although published bioremediation and phytoremediation studies in constructed wetland mesocosms or tailings environment show some promise, their methane reduction efficiencies are generally modest and associated with higher operational costs. For example, bioaugmentation, although it enhanced the efficiency of the phytoremediation system, can only reduce methane emission from an average of 51.3 mg/m^2^/d to 21.6 mg/m^2^/d and from an average of 1708 mg/m^2^/d to 1473 mg/m^2^/d in *Schoenoplectus validus* and *Bambusa vulgaris* horizontal subsurface flow constructed wetland mesocosms.^44^ These reductions remain far less substantial than our results, which were achieved through targeted chemical inhibition. In addition, our study maintains methane inhibition for nearly one year, and likely longer, following a single chemical dosage. Thereby, our results demonstrate that the chemical amendments can substantially inhibit methanogenesis, offering advantages not captured by bioremediation or phytoremediation approaches.

### Molecular Level Characterization of Naphthenic Acids in Tailings

#### Before Chemical Treatment

High resolution mass spectrometry (HRMS) was applied to the analysis of NAFCs in the extracted tailings samples. Before solvent addition in the mesocosm samples, the concentrations of the NAFCs are ∼500 mg/kg for Mesocosm A and ∼1000 mg/kg for Mesocosm B (as shown in Figure 3). The difference in the concentrations of NAFCs in these two mesocosms is due to the different origins of the tailings as well as the formulation of the mesocosms. After solvent addition until steady methane production, the amounts of NAFCs are increased dramatically for both types of mesocosms to nearly 2000 mg/kg. Paraffinic solvent addition has introduced a higher amount of NAFCs compared to that of naphthenic solvent. It seems that the onset of the methanogenesis process has released more NAFCs from the bituminous materials in the tailings, likely due to hydrolytic dissolution. However, although the concentrations of NAFCs were lower in the tailings before solvent addition, there were more NAFCs available with the higher orders of double bond equivalents (DBE) and larger carbon numbers (Figure 4). This may indicate that hydrolysis of NAFCs mainly focused on the fatty acids and lower carbon number compounds. In addition, the distribution of heteroatoms of NAFCs has changed after solvent addition, and the changes were not affected by the type of solvents used but distinct among two mesocosms. For example, mesocosm A has decreased NO_3_, but an O_3_ group appears after solvent addition; meanwhile, mesocosm B has decreased N_2_O_9_, but O_3_S, N_2_O_2_, and N_3_O_3_ groups appear after solvent addition (Figure S3). This indicates that the different microbial processes may have happened inside the different mesocosms.

**Figure 3:**
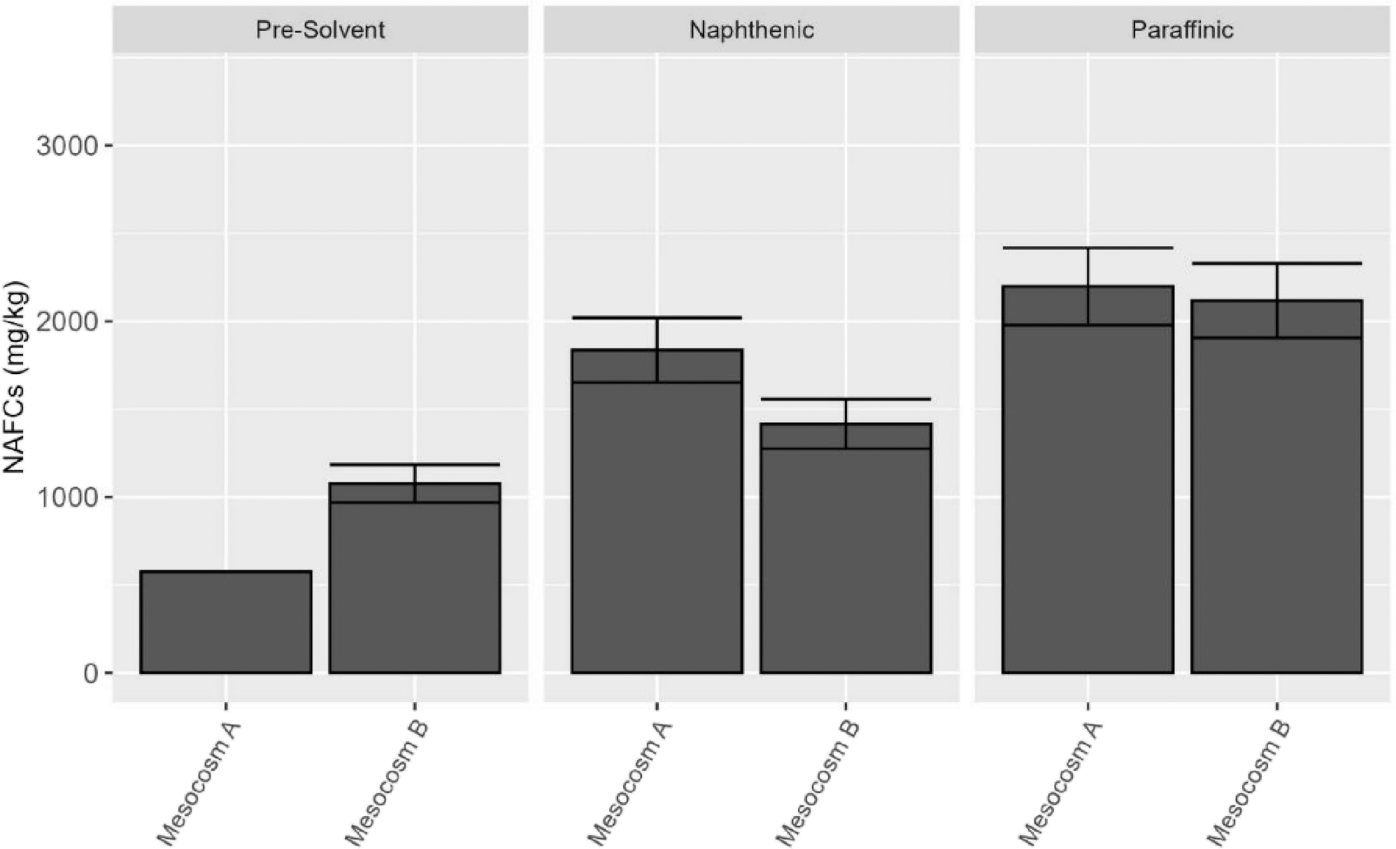
Concentrations of NAFCs in tailings before chemical treatment. Concentrations of NAFCs are reported with the average of duplicate samples, and error bars communicate the range of detected values.

**Figure 4:**
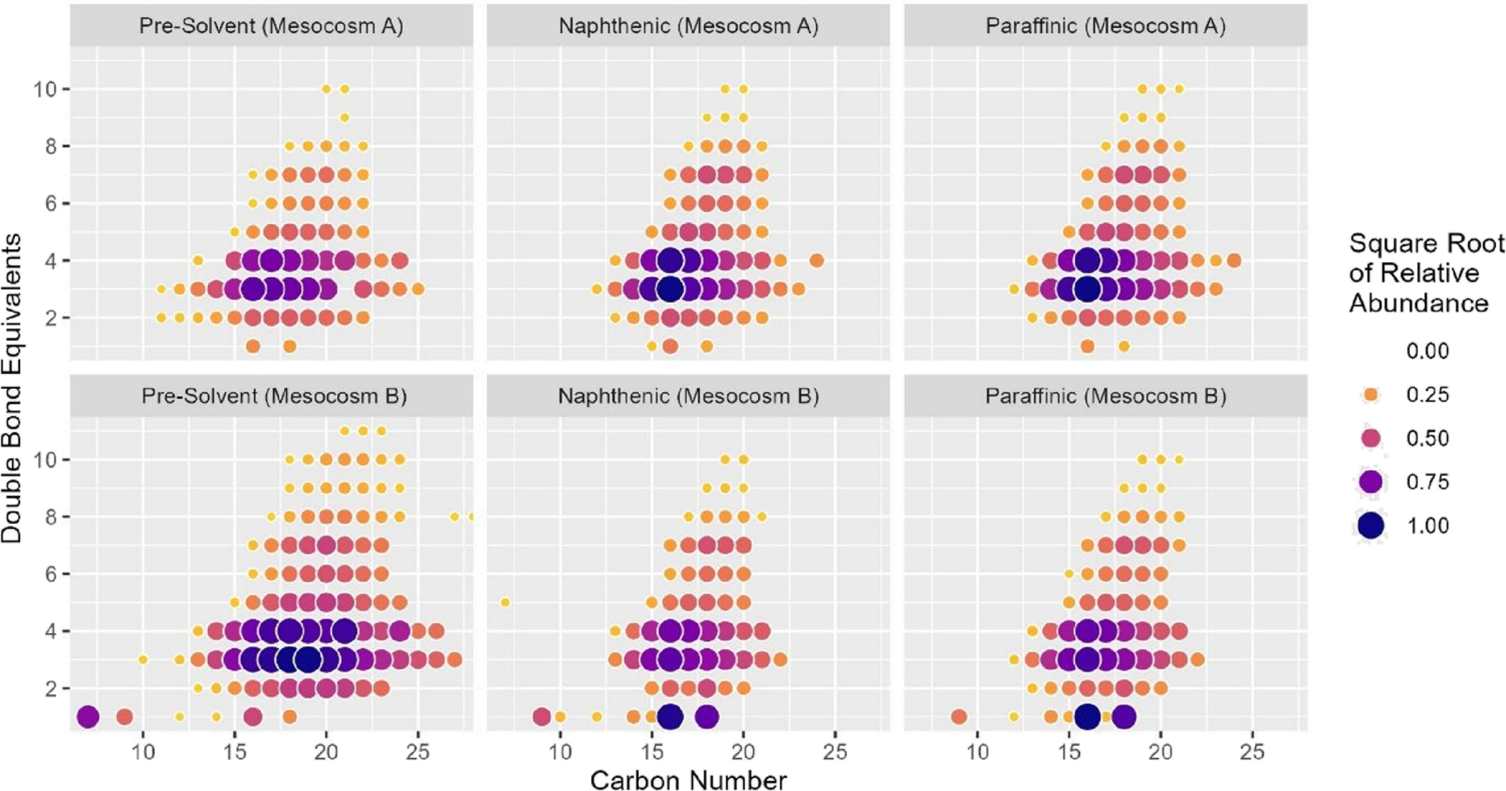
Base peak-normalized abundance of NAFCs in all mesocosms before chemical treatment; *x* axis represents carbon numbers of NAFCs indicating molecular size or chain length, and *y* axis represents double bond equivalents of NAFCs reflecting the degree of unsaturation and ring structures.

#### After Chemical Treatment

After chemical treatment, the concentrations of NAFCs were greatly reduced across all of the bottle tests (Figure 5) compared to the samples before treatment (Figure 3), which indicates that the organic matter degradation process happened during the methanogenesis process despite the applications of various chemical treatments tested in this study. Furthermore, the results show that Fe_3_(SO_4_)_2_ not only inhibited methane emissions but also enhanced the attenuation of NAFCs (Figure 5 and Table S1). This trend was affected by neither the solvent used nor the source of the tailings. The depletion of NAFCs in phase 2 was more dramatic than that in phase 3 of the study, likely due to the consumption of the treatment chemicals with time. However, unlike Fe_3_(SO_4_)_2_, Na_2_SO_4_ did not result in greater reduction of NAs in all cases compared to the control samples. This suggests that Fe^3+^ or iron-reducing bacteria play a key role in enhancing NAFC attenuation. Although SO_4_^2–^ alone can inhibit methane production, Fe^3+^ supports complementary beneficial outcomes.

**Figure 5:**
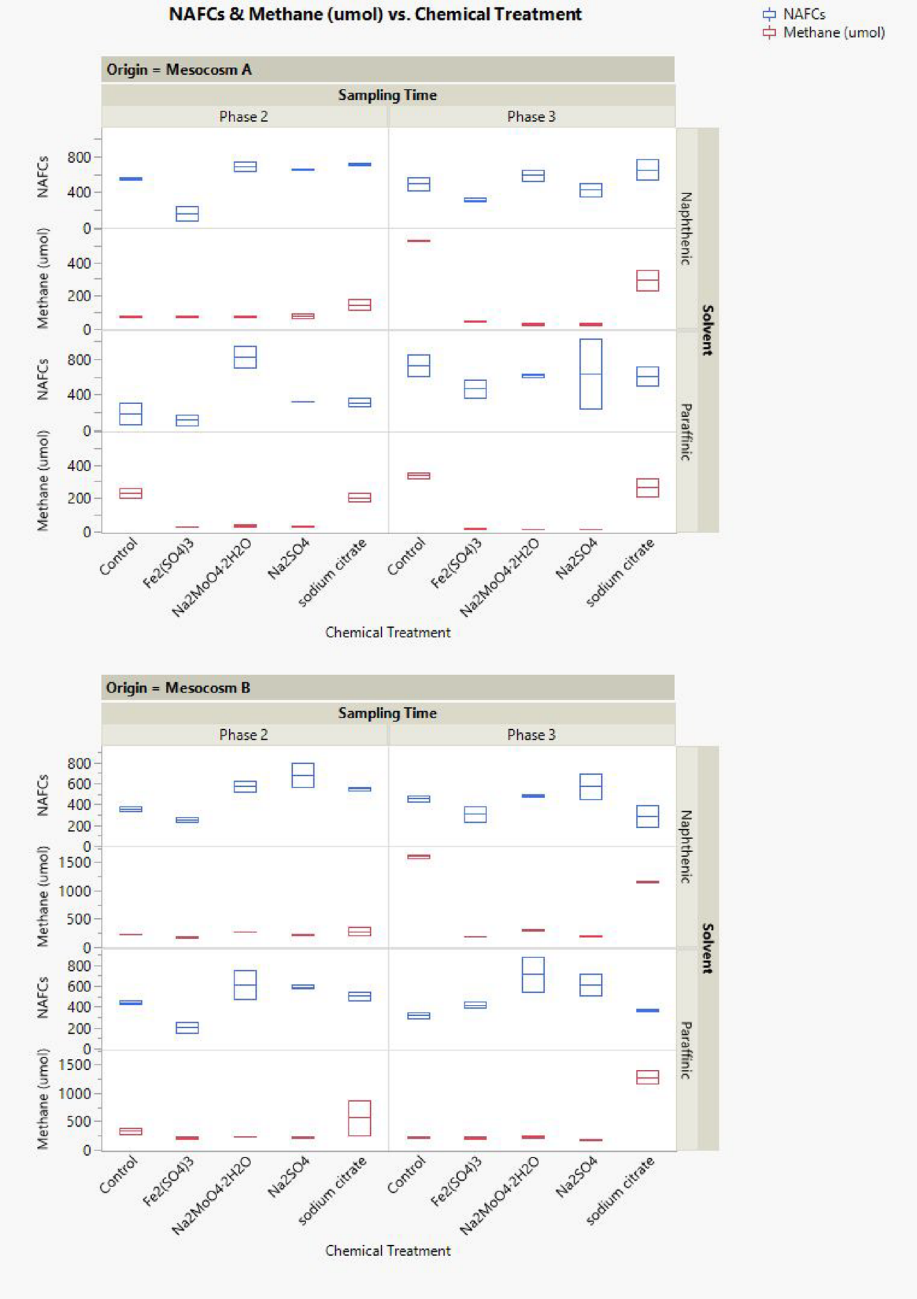
Total NAFCs (mg/kg) in the mesocosm samples and methane content (μmol) in the headspace of the sampling bottles in phases 2 and phase 3. Concentrations of NAFCs are reported with the average of duplicate samples, and error bars communicate the range of detected values.

A previous study demonstrated that anaerobes with metabolic activities related to anaerobic digestion ultimately produced acetate, CO_2_, and H_2_. However, a faster and complete degradation of NAs or other organics is less likely unless aerobic conditions are engineered within the systems.^45^ In our study, based on HRMS results, samples were generally dominated by the strong presence of O_2_-formulas (Figure 6). It also appears that the concentrations of NAFCs were higher in the samples after Na_2_MoO_4_ treatment for both naphthenic tailings and paraffinic tailings. Since both Na_2_MoO_4_ and Fe_2_(SO4)_3_ decreased the methane production, there appears to be no causal relationship between the amounts of NAFCs and methane gas production. However, the fact that Fe_2_(SO_4_)_3_ can reduce both the methane production and the concentration of NAFCs indicates the advantages of the application of this chemical over other treatment chemicals in the tailings pond.

**Figure 6:**
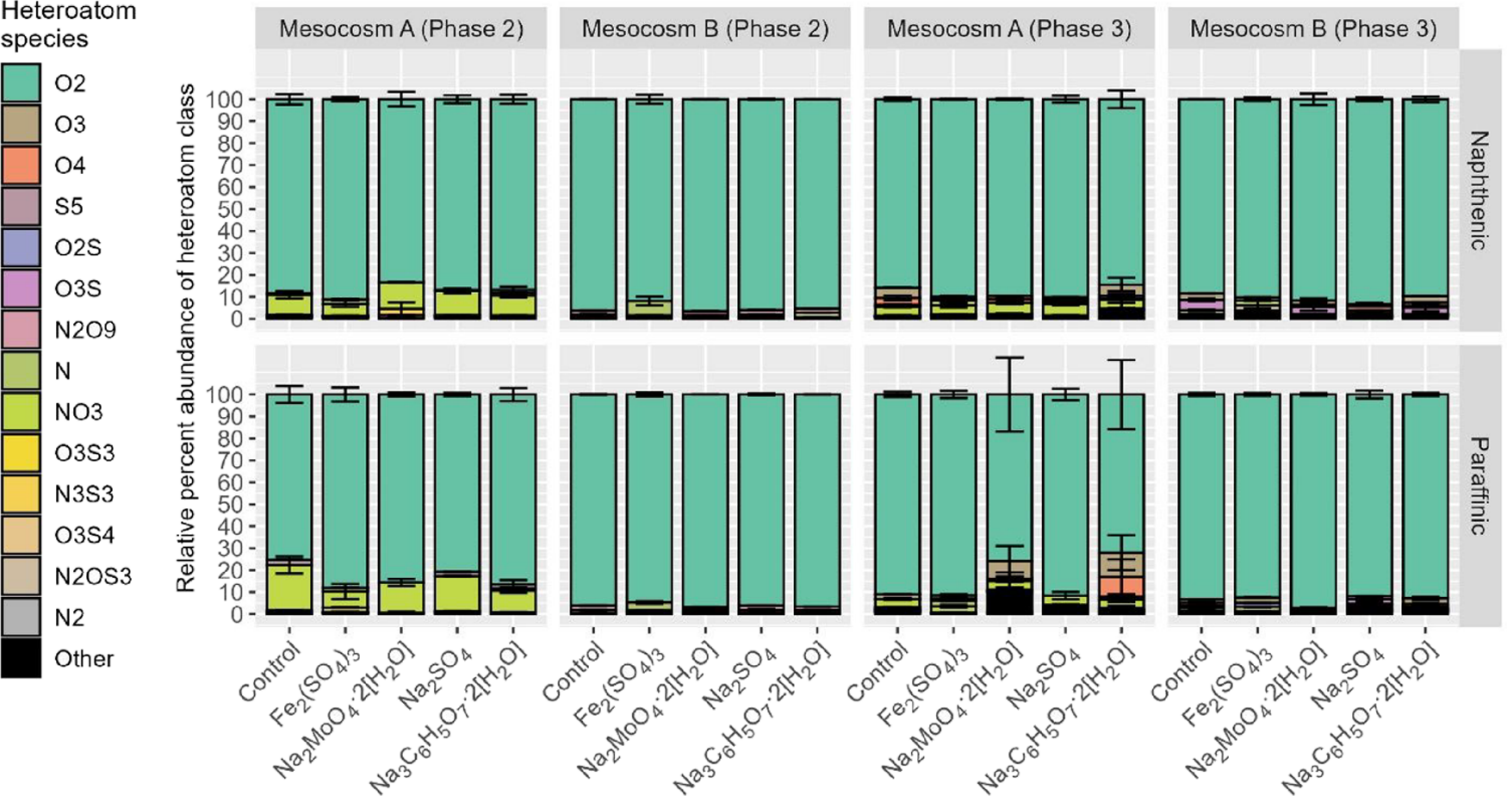
Heteroatoms of NAFCs in the mesocosm samples at different sampling times, phase 2 (February 2023) and phase 3 (Nov. 2023). Spectral formula abundance was dominated by the O_2_-NAFC formulas. Concentrations of NAFCs are reported with the average of duplicate samples, and error bars communicate the range of detected values.

In addition, from phase 2 to phase 3 of the sample monitoring, there was an increase in the higher order of oxidized NAFCs and a reduction of the NO_3_ compounds for Mesocosm A samples, whereas there was an increase of sulfur containing species for Mesocosm B samples (Figure 6). Overall, there were more diverse heteroatomic NAFC species (i.e., non-O_2_ NAFCs) at the end of phase 3 monitoring compared to phase 2 monitoring, which were not affected by the source of tailings or the solvent employed. Furthermore, principal component analysis (PCA) was applied to the spectral data from NAFCs detected in the treated mesocosm samples (Figure 7). High-resolution mass spectral sample data separated on the PCA biplot mostly according to the sampling chronology, distinguishing earlier and later samples, whereas PCA did not clearly distinguish different treatment types or tailings sources from one another. As shown in Figure S4, O_2_-NAFCs with a high carbon number associated with “low” (i.e., solids-like, NA-rich) PC1 coordinates (corresponding to earlier sampling dates), where “high” PC1 coordinates (corresponding to later sampling dates) are associated with the NAFCs with O_3_- and O_4_-NAFCs, as well as with O_2_-NAFCs with a lower carbon number. In addition, the O_2_-class compounds show the greatest structural diversity, while the O_3_ and O_4_ compounds exhibit higher DBE values and cluster toward higher PC1 scores, potentially reflecting increased aromaticity, oxygenation, or molecular complexity. This indicates the dynamics of different processes in the mesocosms. Methanogenesis and chemical inhibition process will likely degrade the NAFCs with high carbon numbers. Moreover, chemical treatment enhances the degradation process. However, once the chemicals get consumed, hydrolysis of NAFCs will reintroduce the fatty acids and lower carbon number compounds back into the system.

**Figure 7:**
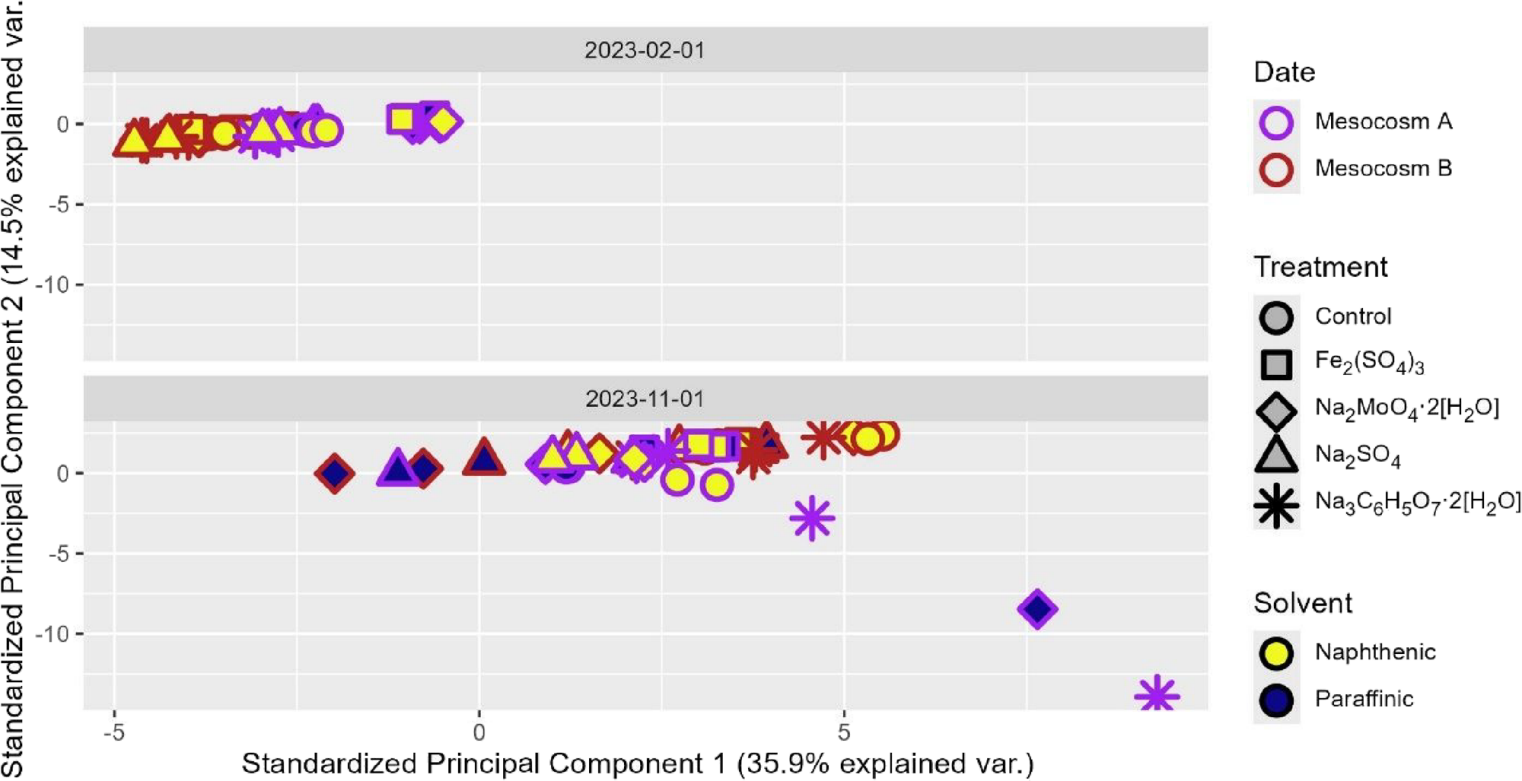
Principal component analysis (PCA) of all mesocosm samples collected over the course of the present study was based off a Pareto-normalized PCA of base peak-standardized formula abundance. Phase 2 is represented by 2023–02–01, and phase 3 is represented by 2023–11–01. Interior color denotes the solvents added, exterior color denotes mesocosms A and B, and shape denotes the treatments.

As O_2_-NAFCs are implicated as primary toxicants, we examined the O_2_-NAFCs by comparing different treatment methods using two solvents with two types of tailings during different phases of the monitoring periods. As shown in Figure S5–S8, from phase 2 to phase 3, in general, a decrease in the spectral abundance of higher-C# O_2_-NAFCs was observed as suggested by PCA eigenvectors. This shows that degradation of the NAFCs occurred under all the experimental conditions tested in this study, highlighting the microbial consortia’s ability to degrade the O_2_-NAFC species. The decreases in spectral abundance were centered among the highest weight saturated formulas between C# 15 to 25, while those formulas increasing in relative spectral abundance tended to be lighter-molecular weight unsaturated (DBE 4–10, increasing with C#) formulas from C# 10 to 18 (as shown in Figures S9 and S10). Such shifts in molecular features might reasonably be anticipated to correspond to lesser toxicity as a result of lesser lipophilicity, following principles laid out by Scarlett et al.^46^ and Hughes et al.^47^ It appears that the ferric/sulfuric conditions in combination with an actively methanogenic microbial community have the potential for selective biodegradation of heavier (i.e., a higher number of carbon atom) O_2_ species. There was also a subtle difference in terms of the distribution of the O_2_ species among different treatment methods for Mesocosm B samples. On the other hand, in Mesocosm A samples, the distribution of O_2_ species after Na_2_MoO_4_ treatment in phase 2 had a narrower range of carbon # and DBE # compared to the other treatment methods in the same monitoring phase, while the distribution after Fe_2_(SO_4_)_3_ treatment in phase 3 had a narrower range of carbon # and DBE #. This distribution pattern was not affected by the type of solvent used in the experiments.

A previous study investigated anaerobic biodegradation of 5 single-ringed surrogate NAs or acid-extractable organics in enrichment cultures established from anoxic tailings under nitrate-, sulfate-, iron-reducing, and methanogenic conditions.^48^ Simpler surrogate NAs can be biodegraded under a variety of anoxic conditions, and more complex NAs (in the form of acid-extractable organics) were observed to drive the reduction of sulfate and iron relative to controls. Surrogate NA depletion was observed under all anaerobic conditions tested to varying extents, correlating to losses in the respective electron acceptor (sulfate or nitrate) or the production of predicted products (Fe­(II) or methane). Unfortunately, the iron-reducing conditions in the previous study were compromised by citrate additions, which changed the microbial environment from iron-reducing to methanogenic. Our study has provided additional information on a related subject by taking a detailed look at the NAFCs using high resolution mass spectrometry. Overall, our study shows that the combination of sulfate-reducing conditions and iron-reducing conditions may have superior performance with respect to both degrading NAFCs, thereby likely mitigating toxicity, while also jointly inhibiting methane emissions in the oil sands tailings ponds. However, it is to be noted that certain cations may affect the solubility, partitioning or ionization of the NAFCs,^49^ which may or may not affect our experimental results. However, in our attempted investigation, we found that the cation effect was negligible for the concentration changes of NAFCs using the treatment chemicals in this study.

In reality, environmental factors critically mediate the success of chemical and biological strategies for NAFC reduction. Temperature governs both reaction kinetics and microbial activity, making seasonality a major determinant of degradation rates in oil sands environments. Elevated salinity, typical of process-affected waters, can suppress microbial diversity and scavenge oxidative radicals, thus reducing chemical treatment efficiency. Oxygen availability dictates dominant metabolic pathways, with aerobic conditions favoring rapid oxidative breakdown and anoxic zones fostering recalcitrant intermediates and methane generation. Consequently, the optimization of NAFC treatment requires an integrated approach that aligns amendment chemistry, microbial ecology, and environmental conditions.

Recall that in part I of this two-article publication series,^27^ Spirochaetaceae and Thermovirgaceae were increased in abundance at the end of phase 3 methane monitoring across all the samples and Geobacteraceae was abundant in the Fe_2_(SO_4_)_3_-treated samples. Since Thermovirgaceae are capable of fermentation or synergistic hydrocarbon degradation^50,51^ and Spirochaetaceae generally contribute to necromass recycling in anoxic hydrocarbon-contaminated environments,^52^ we believe that the degradation of NAFCs under different chemical treatment conditions was induced by a combination of multiple bacteria. Under Fe_2_(SO_4_)_3_ treatment, it is possible that Thermovirgaceae, a group of Clostridia, may directly degrade complex organics including NAs, while Geobacteraceae may assist through syntrophic interactions or cometabolism under iron-reducing conditions. For other treatment conditions and control experiments, it is likely that sulfate-reducing or fermenting bacteria primarily mediate the anaerobic transformation of hydrocarbon compounds in the early stage, but Thermovirgaceae is responsible for the later stage of hydrocarbon degradation with the aid of Spirochaetaceae. Literature also shows that several microorganisms have been successfully isolated from OSPW or other NA-contaminated environments and were capable of degrading NAs. These include the genera *Pseudomonas*, *Alcaligenes*, *Acinetobacter*, *Kurthia*, *Rhodococcus*, *Acidovorax*, and *Rhodoferax*.^53–56^ However, these microbes were below the detection limit in our mesocosms. Further investigation is needed to determine which microbes are the major drivers for NA degradation and transformation during the methane inhibition process.

It is well established that most microorganisms degrade aliphatic and alicyclic carboxylic acids through the β-oxidation pathway,^48,57^ while the benzoate metabolism pathway also plays a key role in the biodegradation of alicyclic carboxylic acids.^58^ In addition, under anaerobic conditions, hydrocarbon degradation can proceed efficiently when methanogens are present.^59^ Using RNA sequencing, previous studies have proposed syntrophic mechanisms in which hydrocarbon or NA degraders transfer electrons to methanogens via intermediates such as H_2_ or formate, and H_2_ is subsequently utilized by hydrogenotrophic methanogens for CH_4_ production.^45^ Given the simultaneous occurrence of multiple biogeochemical processes in our mesocosms and the complexity of the NAFC mixtures, the specific chemical pathways involved could not be directly resolved in this study. Nonetheless, the pathways described above are likely contributors to NAFC attenuation. Future work using model NAFC compounds and controlled biochemical assays will be needed to elucidate the detailed degradation mechanisms.

While Fe_2_(SO_4_)_3_ chemical treatments effectively suppress greenhouse gas emissions and accelerate the degradation of NAFCs, they can impose lasting impacts on microbial community composition and function within tailings ponds. Strong oxidants and metal-based amendments often exert broad selective pressures, reducing microbial diversity and altering key metabolic pathways such as methanogenesis, sulfate reduction, and hydrocarbon degradation. These shifts may simplify community networks and compromise long-term biogeochemical stability. Although partial recovery of indigenous populations can occur once treatment intensity declines, functional profiles often remain distinct from untreated systems, reflecting legacy effects on microbial metabolism and gene expression. Sustainable tailings management therefore requires integrating chemical and biological approaches, using environmentally compatible reagents, allowing recovery intervals and monitoring microbial functionality, to ensure that short-term remediation gains do not undermine the long-term ecological resilience and self-purification capacity of reclaimed aquatic environments.

### Dean–Stark Analysis

Due to limited sample quantity, Dean–Stark analysis was only performed on the Mesocosm B samples at the end of the monitoring period when the bottles were dissembled. As shown in Figure 8, the percentage of extract (e.g., bitumen) for paraffinic tailings was higher than that for naphthenic tailings. There was a statistically significant difference between groups as determined by one-way ANOVA (F­(1,18) = 32.7317, *p* < 0.0001), as shown in Figure S11. Comparison for each pair using Student’s *t* test was also performed on the sample means, and the results demonstrate that pairs of means are significantly different, which indicates more bitumen was consumed during the bottle test when naphthenic solvent was present compared to the presence of paraffinic solvent. Assuming that the solid and water contents in the mesocosms remain unchanged during the methane production process, the decrease in the extract content would be due to the consumption of bitumen during the methane production or inhibition process. This may indicate that during methanogenesis and chemical treatment, the microbial community digests not only labile hydrocarbons but also complex organic matter in the tailings. Faster reactions happened in the naphthenic mesocosms, leading to a low extract percentage in the tailings compared to the paraffinic mesocosms.

**Figure 8:**
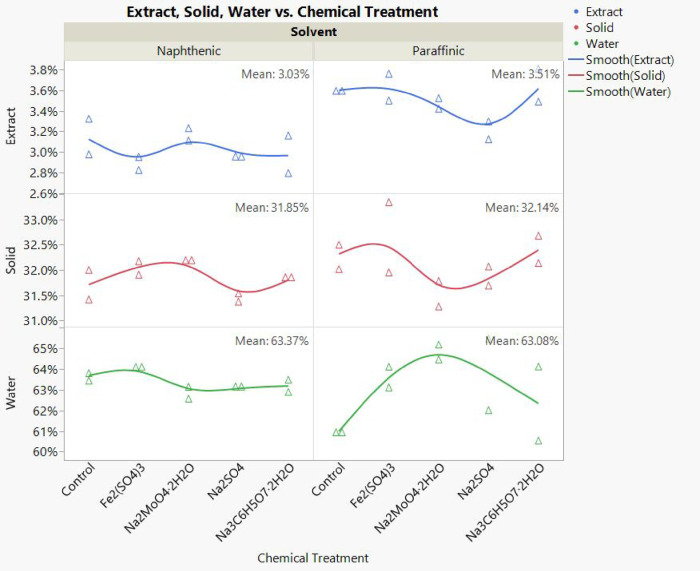
Dean–Stark analysis for Mesocosm B tailing samples (sampled at the end of phase 3). Weight percents of extract, solid, and water are shown by the *y* axis.

Nevertheless, there was no clear evidence of the impacts of different chemical treatments on the compositions of tailings. As shown in Figure S12, a significant difference was only observed between limited chemical treatment groups (Na_2_SO_4_ versus Fe_2_(SO_4_)_3_, control and sodium citrate) for the amount of extract in paraffinic tailings samples as determined by each pair Student’s *t* test (*p* < 0.05). The reason for the difference between Na_2_SO_4_ and the three other groups (i.e., Fe_2_(SO_4_)_3_, control and sodium citrate) remains unclear and needs further investigation. In addition, there was no statistical difference among the treatment groups for naphthenic mesocosm samples, although Fe_2_(SO_4_)_3_ seems to lower the bitumen amount slightly compared with the other treatment chemicals.

### Microtox Analysis

Due to the limited quantity in the surface water, pore water was collected for Microtox analysis to screen sample toxicity with or without chemical treatments. As shown in Figure 9, the Microtox data from samples after Fe_2_(SO_4_)_3_ treatment indicated little-to-no toxicity, except for the Mesocosm A naphthenic samples, which require some follow-up testing. One-way ANOVA analysis was applied to investigate the Microtox data. As shown in Figure S13, it appears that the decrease in the toxicity of the pore water after Fe_2_(SO_4_)_3_ treatment was statically significant (*p* < 0.001). Therefore, it seems that Fe_2_(SO_4_)_3_ treatment lowered the toxicity of the pore water compared with the control samples. Other treatment methods resulted in similar or higher pore water toxicity compared to control samples. However, it is generally believed that the Microtox test is a screening tool and has some limitations.^60,61^ Therefore, it is recommended to conduct a more comprehensive toxicity test if these treatment methods will be applied on a larger scale in the future.

**Figure 9:**
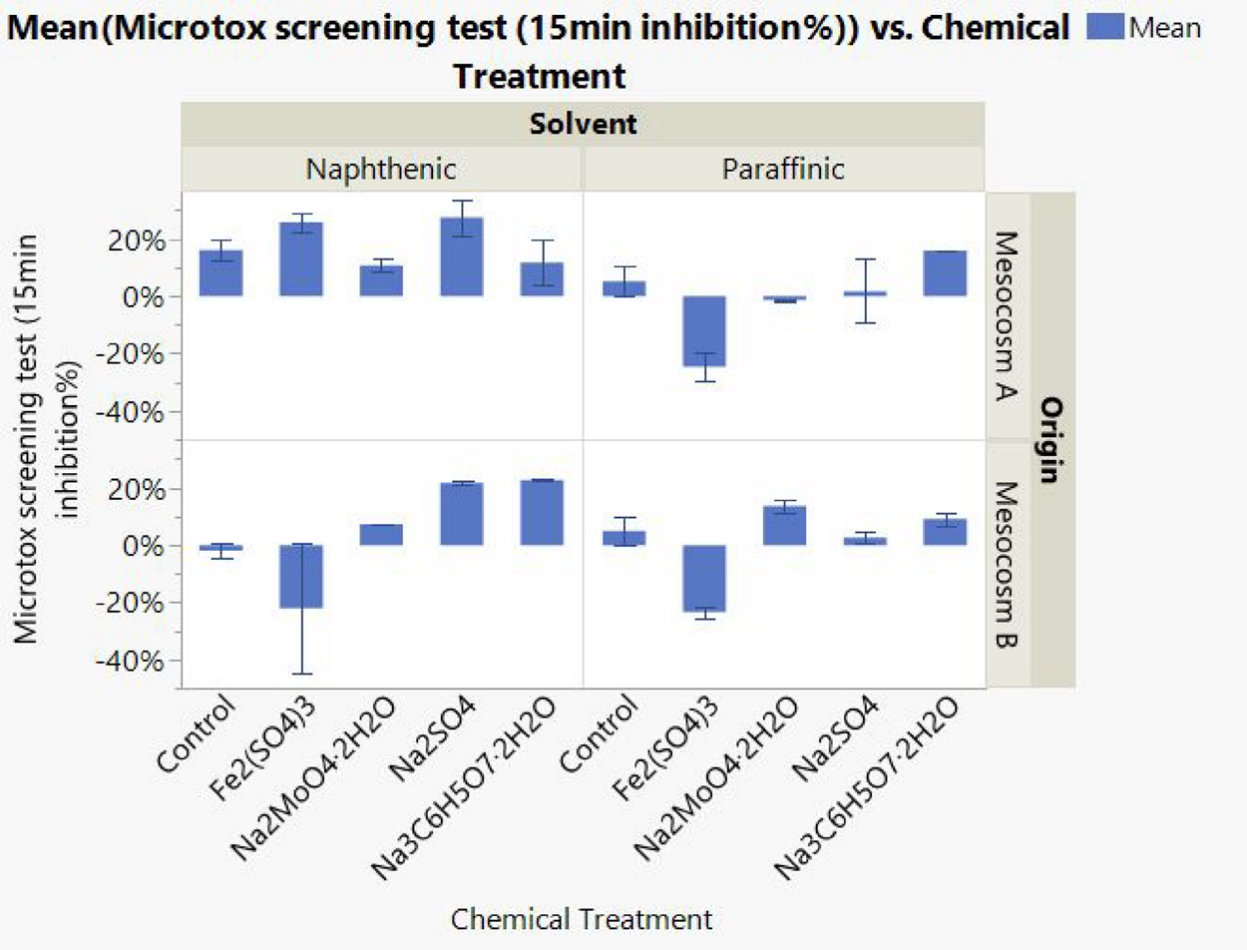
Inhibition% of Microtox analysis at 15 min for pore water samples following various chemical treatments. Each error bar is constructed using the minimum and maximum of the data.

### Implications for Environmental Safety of Chemicals in Industrial-Scale Applications

While the use of different chemical amendments represents an innovative approach to mitigating methane emissions, a detailed assessment of cost-effectiveness and environmental safety in industrial-scale applications would further strengthen the practical significance of this work. Part I of the study has already provided large-scale cost estimates for the treatment chemicals.^27^ In the present paper, we focus on evaluating the environmental safety of the three chemicals (i.e., Na_2_MoO_4_·2H_2_O, Fe_2_(SO_4_)_3_, and Na_2_SO_4_) that demonstrated the capability of methane inhibition in the tailings environment.

Acute and chronic toxicity data for sodium molybdate (Na_2_MoO_4_·2H_2_O) have been reported for several aquatic species. For *Daphnia magna*, the 48-h LC50 value was 367.8 mg L^–1^, while chronic EC10 values were 62.8–105.6 (mg Mo)/L based on 21 day-reproduction.^62,63^ For other sensitive species (such as rainbow trout, snail, and frog), the reported EC10 values range from 43.2 to 241.5 mg/L at various monitoring periods.^63^

However, there seems to be limited toxicity data for Fe_2_(SO_4_)_3_; therefore, Fe^3+^ and sulfate must be evaluated separately. U.S. EPA has established a chronic criteria value for freshwater aquatic life as 1.0 mg/L for iron.^64^ In particular, for invertebrates, *Asellus aquaticus* exhibited LC50 of ∼183 mg Fe/L at 48 h exposure and ∼124 mg Fe/L at 96 h exposure under certain conditions.^65^ For chronic effects, 5.2 mg/L Fe^3+^ induced 50% reproductive impairment for *Daphnia magna*.^66^ Whereas sulfate toxicity varies widely with water chemistry, most tested organisms tolerate relatively high concentrations. Across species, chronic EC10 or LC10 values typically range from 926 to 1387 mg SO_4_^2–^/L. However, the cladoceran *Daphnia longispina* and freshwater snail *Lymnaea stagnalis* were more sensitive, with reproductive effects at 49 mg SO_4_^2–^/L and growth inhibition at 217 mg SO_4_^2–^/L, respectively.

For sodium sulfate, species-dependent chronic effects were reported at hundreds to thousands of mg/L in organisms such as *Ceriodaphnia dubia*, *Chironomus*, *Lampsilis*, and *Pimephales*. Acute LC50 values for fish generally exceed 1000 mg/L.^67^

Overall, the toxicity data suggest that, aside from sodium sulfate, both sodium molybdate and ferric sulfate exhibit moderate toxicity to aquatic species, given the quantity of treatment chemicals applied in this study. However, it is noted that toxicity strongly depends on water chemistry since it controls the bioavailability and speciation of metals. Importantly, following chemical treatment, Mo and Fe concentrations are expected to decrease substantially due to precipitation, sorption, and incorporation into mineral phases. Therefore, the optimal dosage of the chemical treatment should be explored in future work to ensure environmental safety and reduce operational cost. The dosages used in this study can be considered conservative, representing a high-end scenario to demonstrate treatment feasibility. Long-term environmental monitoring is also important for the safe deployment of these chemicals in large scale industrial applications. Nevertheless, chemical amendments can reduce methanogenesis in tailings ponds and accelerate the transformation of NAFCs, but they rarely solve every problem alone. Pairing amendments with other environmentally friendly techniques (such as carbon capture, renewable energy, bioremediation, or constructed wetlands) multiply benefits with long-term GHG mitigation, which in turn improves ecological outcomes and strengthens the case for regulatory and financial support in the near future.

## Conclusions

The molecular characteristics of O_2_-NAFCs all shifted with decreases in carbon number but increasing DBEs, consistent with the degradation of NAFCs under all the experimental conditions tested in this study. Furthermore, Fe_2_(SO_4_)_3_ can be used as an effective chemical to both reduce methane production and promote degradation of the NAFCs in the solid phase and thus decrease the overall aquatic toxicity. There were subtle differences among the solvents used and the sources of tailings. Nevertheless, trends were similar for different chemical treatments across the different types of tailings. No causal relationship between the NAFCs and methane production could be proposed based on these data given different chemicals used in this study. This is likely due to the fact that many different microbes were involved in the degradation process, and thus, the impact of the NAFCs from the methanogenesis and its inhibition process was likely to be chemical dependent. This study establishes the importance of integrating sulfate and iron treatments into tailings pond management plans to reduce methane emissions, attenuate NAFCs, and improve water quality.

## Supplementary Material



## Notes

One-way ANOVA analysis of methane emissions by chemical treatment; heteroatoms of NAFCs in the tailings sample before chemical treatment; a carbon number versus double bond equivalents plot showing the relative weightings of different formulas detected in mesocosms; base peak-normalized abundance of O_2_-NAFCs in mesocosms; net-change carbon number versus double bond equivalents plots of O_2_-NAFCs over entire experimental periods; one-way ANOVA analysis of Dean–Stark extract by the type of solvent and by chemical treatment for Mesocosm B; one-way ANOVA analysis of Microtox data by chemical treatment; the percentage changes of the NAFC concentrations for each treatment relative to the control (PDF)
