# Supplementary material for: Mesocosm Study of Chemical Treatments on Methane Emissions in Oil Sands Tailings Ponds – Part II: Illustrating the Relationship of Naphthenic Acids with Methanogenesis
Source: ACS Omega. 2026 Jan 19;11(4):5839–52. doi: 10.1021/acsomega.5c09941 (PMC12878770; doi:10.1021/acsomega.5c09941)
Supplement: Supplementary file 1 [file ao5c09941_si_001.pdf]

Supplementary Information for:

Mesocosm Study of Chemical Treatments on Methane Emissions in Oil Sands Tailings Ponds – Part II: Illustrating the Relationship of Naphthenic Acids with Methanogenesis

Xiaomeng Wang[1], Ian Vander Meulen[2], Dena W. McMartin[3], Chukwuemeka Ajaero[2], John Headley[2], and Bipro Ranjan Dhar[4]*

[1] Natural Resources Canada, CanmetENERGY Devon, 1 Oil Patch Drive, Devon, Alberta T9G 1A8, Canada

[2] Environment and Climate Change Canada, Watershed Hydrology and Ecology Research Division, National Hydrology Research Center, 11 Innovation Boulevard, Saskatoon, Saskatchewan S7N 3H5, Canada

[3] University of Lethbridge, Department of Geography and Environment, 4401 University Drive, Lethbridge, Alberta T1K 3M4, Canada

[4] University of Alberta, Faculty of Engineering, Civil and Environmental Engineering Department, 9211 116 Street, Edmonton, Alberta, T6G 2H5, Canada

*Corresponding Author: xiaomeng.wang@nrcan-rncan.gc.ca

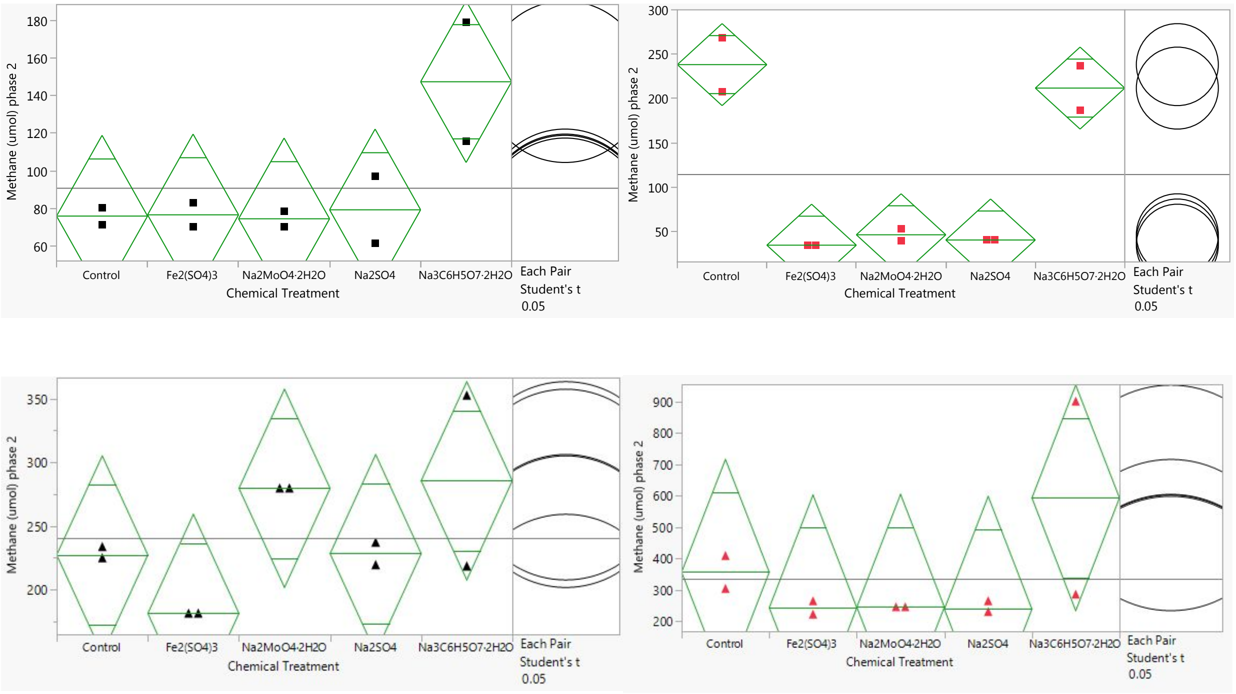

Figure S1 – One-way ANOVA analysis of methane emissions by chemical treatment based on the headspace methane data in phase 2. Mesocosm A naphthenic tailings (top left, solid black square) and Mesocosm A paraffinic tailings (top right, solid red square); Mesocosm B naphthenic tailings (bottom left, solid black triangle) and Mesocosm B paraffinic tailings (bottom right, solid red triangle)

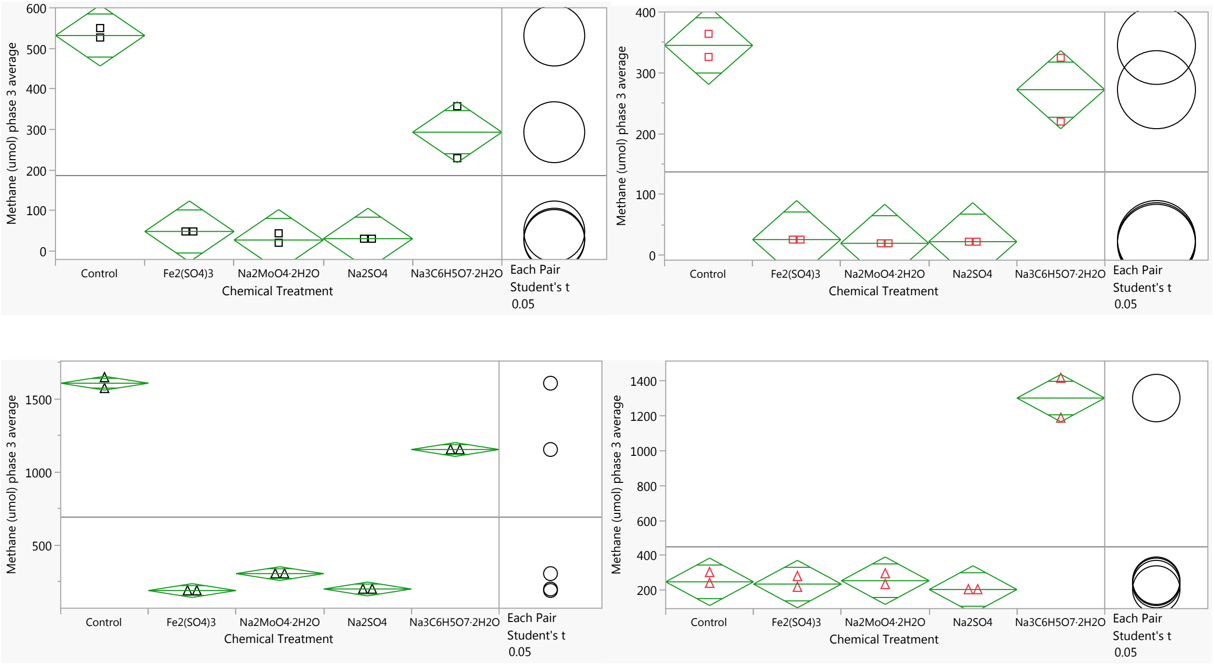

Figure S2 – One-way ANOVA analysis of methane by chemical treatment based on the averaged phase 3 headspace methane data in Mesocosm bottles. Mesocosm A naphthenic tailings (top left, open black square) and Mesocosm A paraffinic tailings (top right, open red square); Mesocosm B naphthenic tailings (bottom left, open black triangle) and Mesocosm B paraffinic tailings (bottom right, open red triangle)

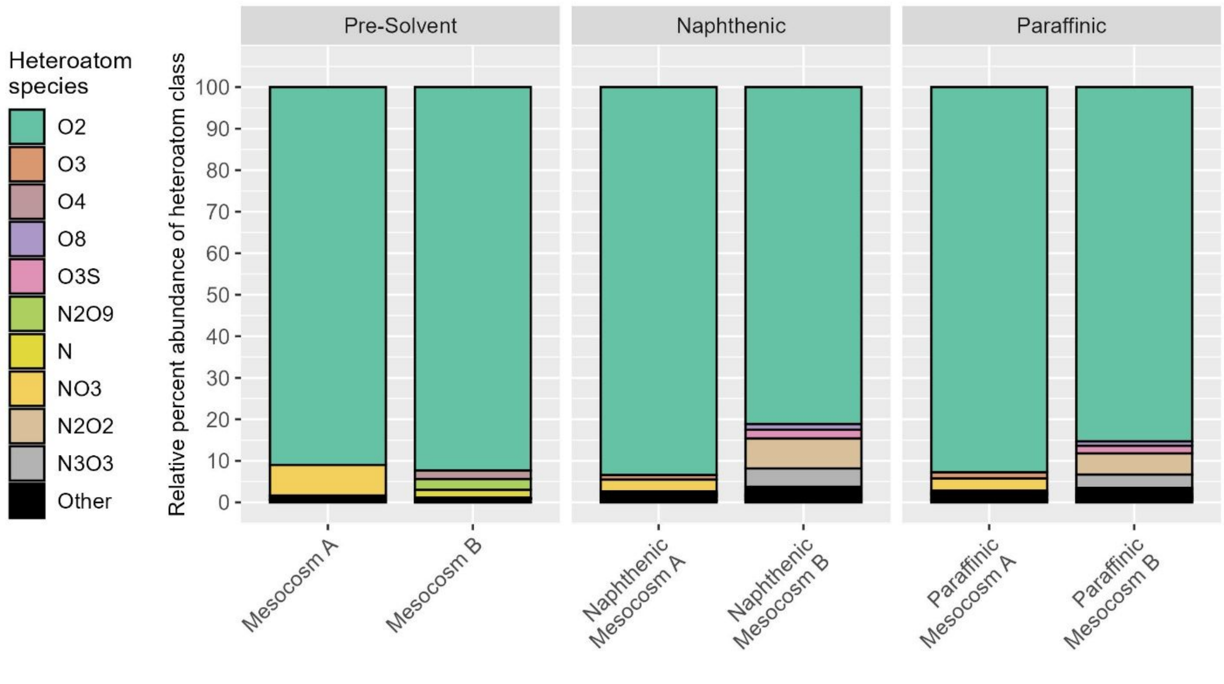

Figure S3 – Heteroatoms of NAFCs in the tailings sample before chemical treatment

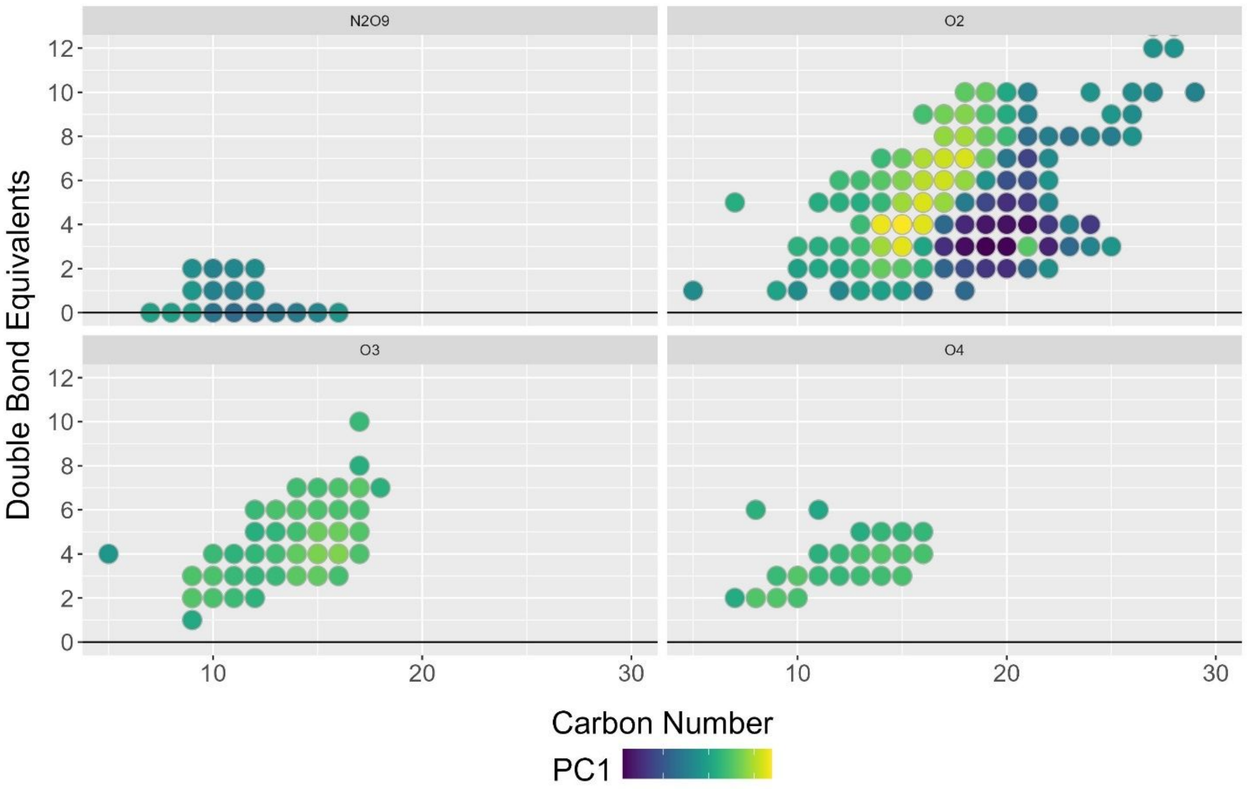

Figure S4 – A carbon number versus double bond equivalents plot showing the relative weightings of different formulae detected in tailings mesocosms, as evaluated by a Pareto-normalized principal components analysis of base peak-standardized formula abundance. Point color corresponds to Principal Component 1 (PC1) scores, with the color gradient from purple (low PC1) to yellow (high PC1) indicating relative differences in underlying chemical properties.

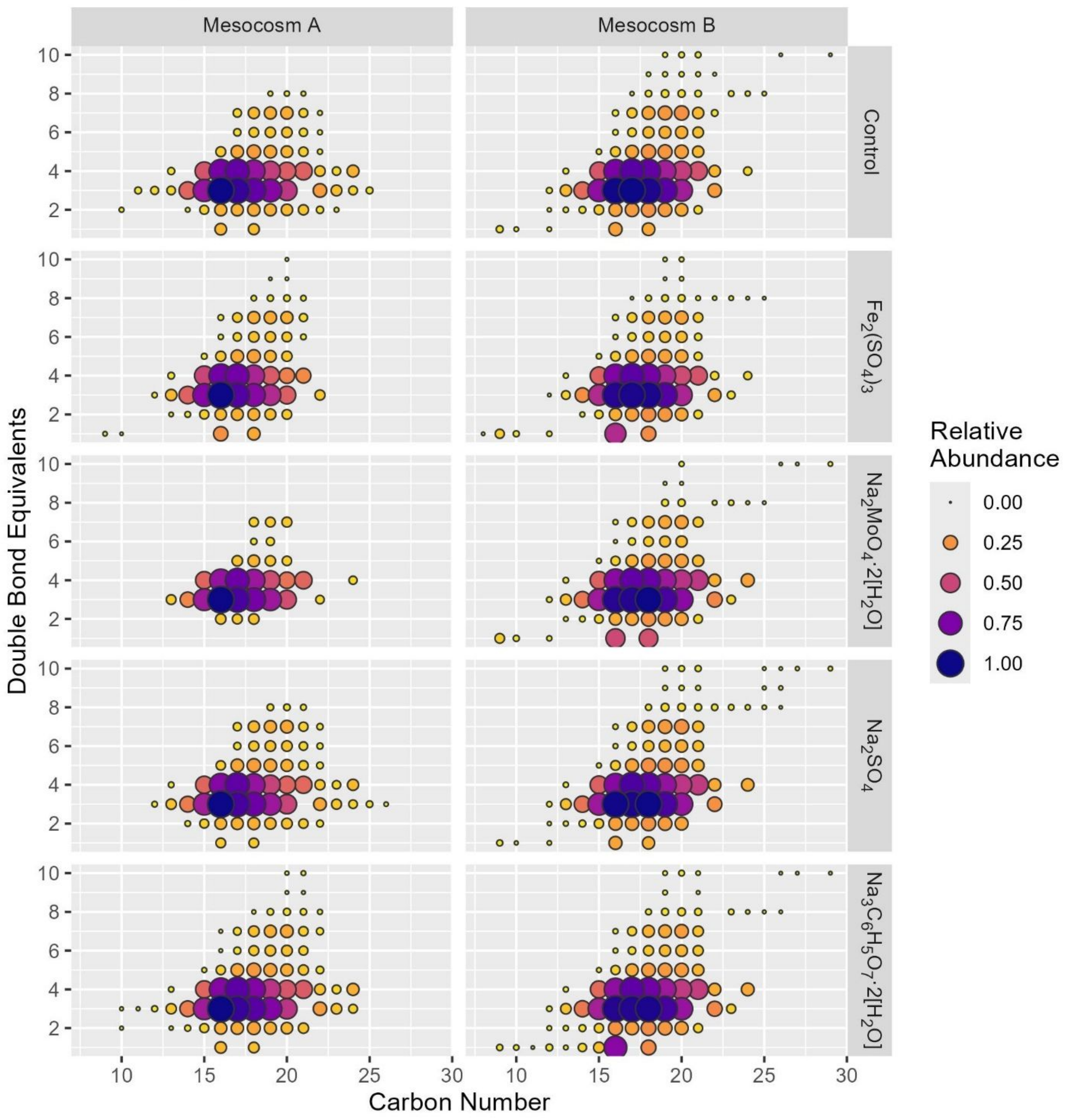

Figure S5 – Base peak-normalized abundance of O_2-NAFCs in mesocosms supplemented with paraffinic solvents in phase 2.

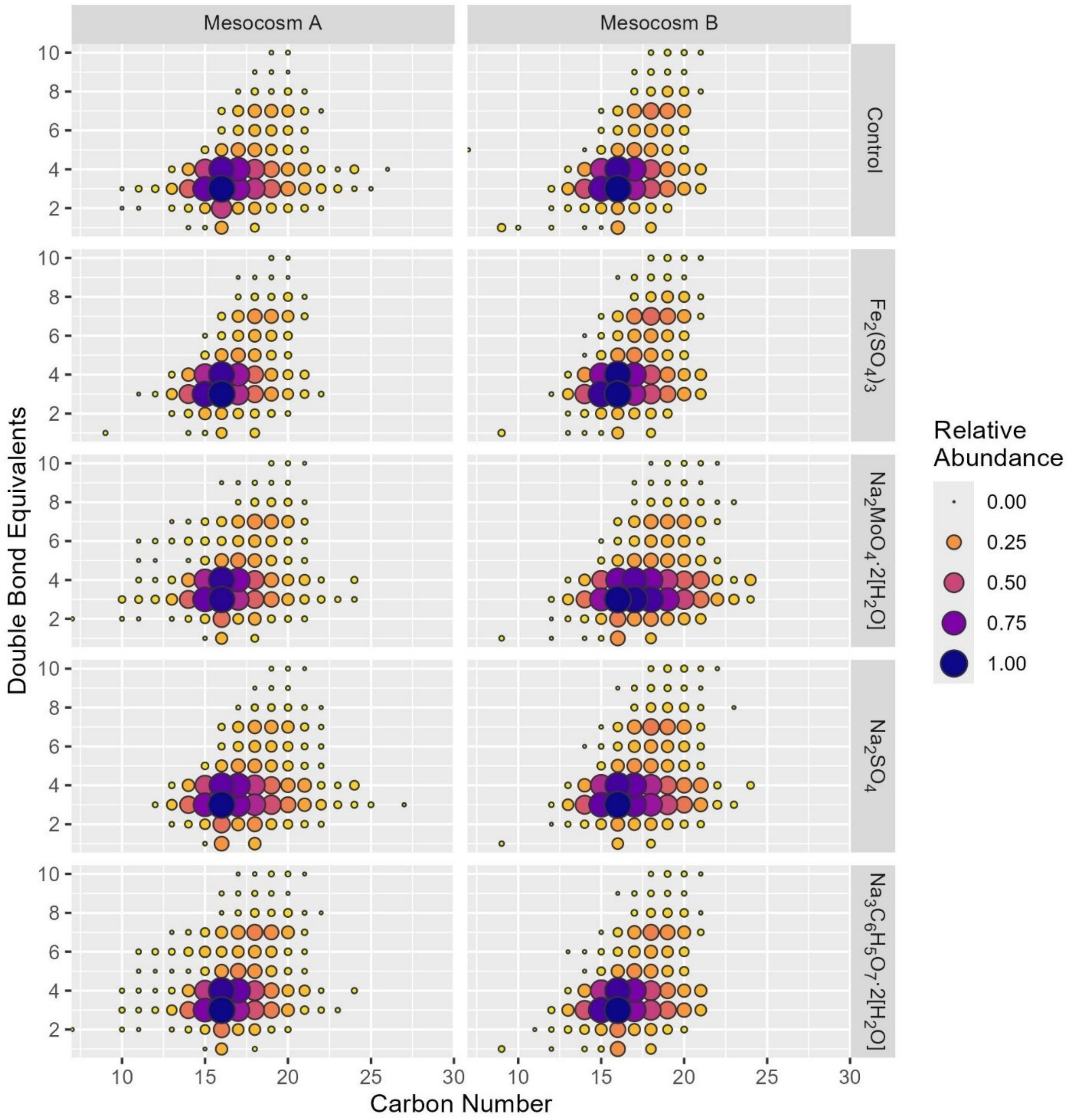

Figure S6 – Base peak-normalized abundance of O_2-NAFCs in mesocosms supplemented with paraffinic solvents in phase 3.

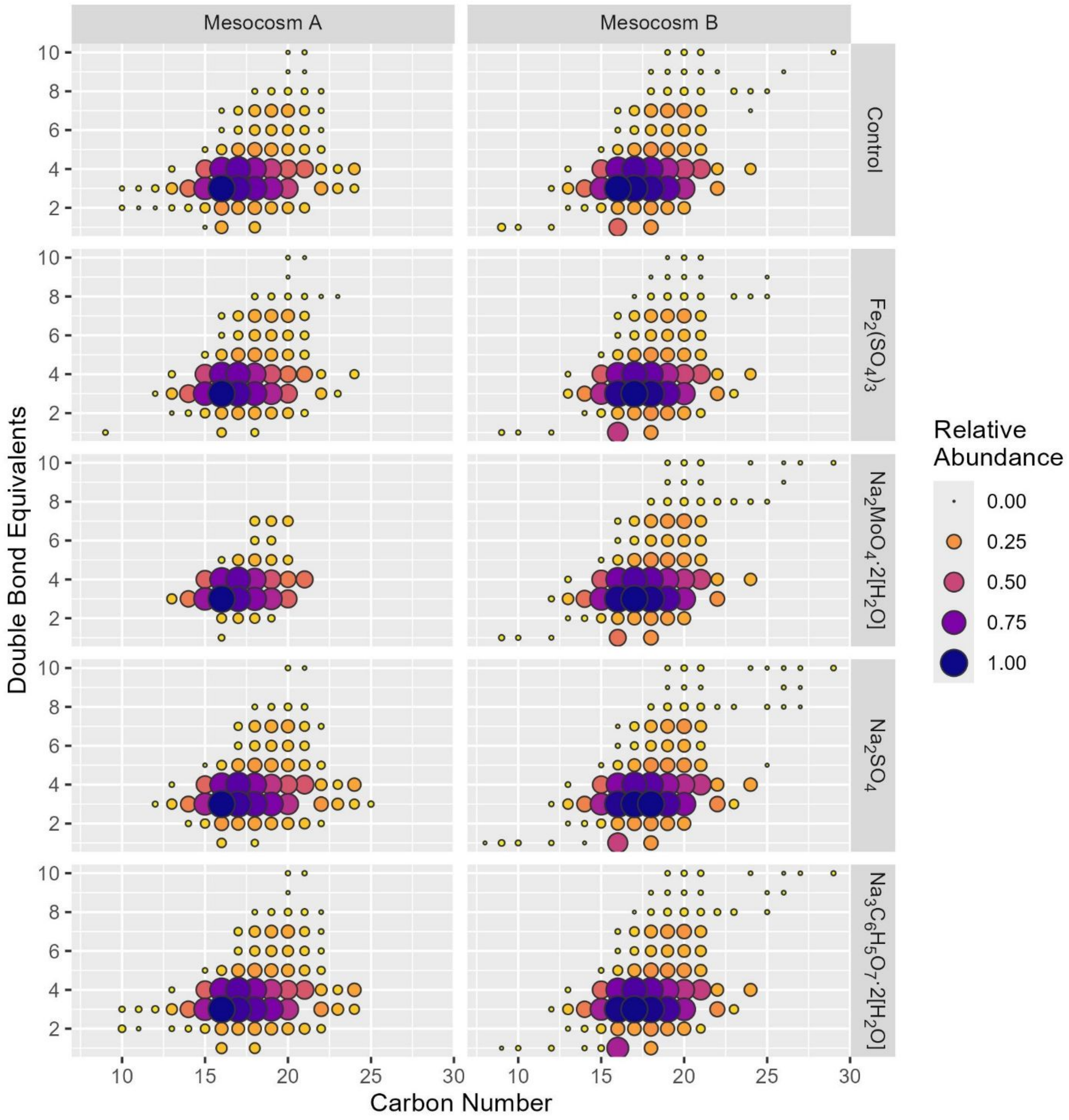

Figure S7 – Base peak-normalized abundance of O_2-NAFCs in mesocosms supplemented with naphthenic solvents in phase 2.

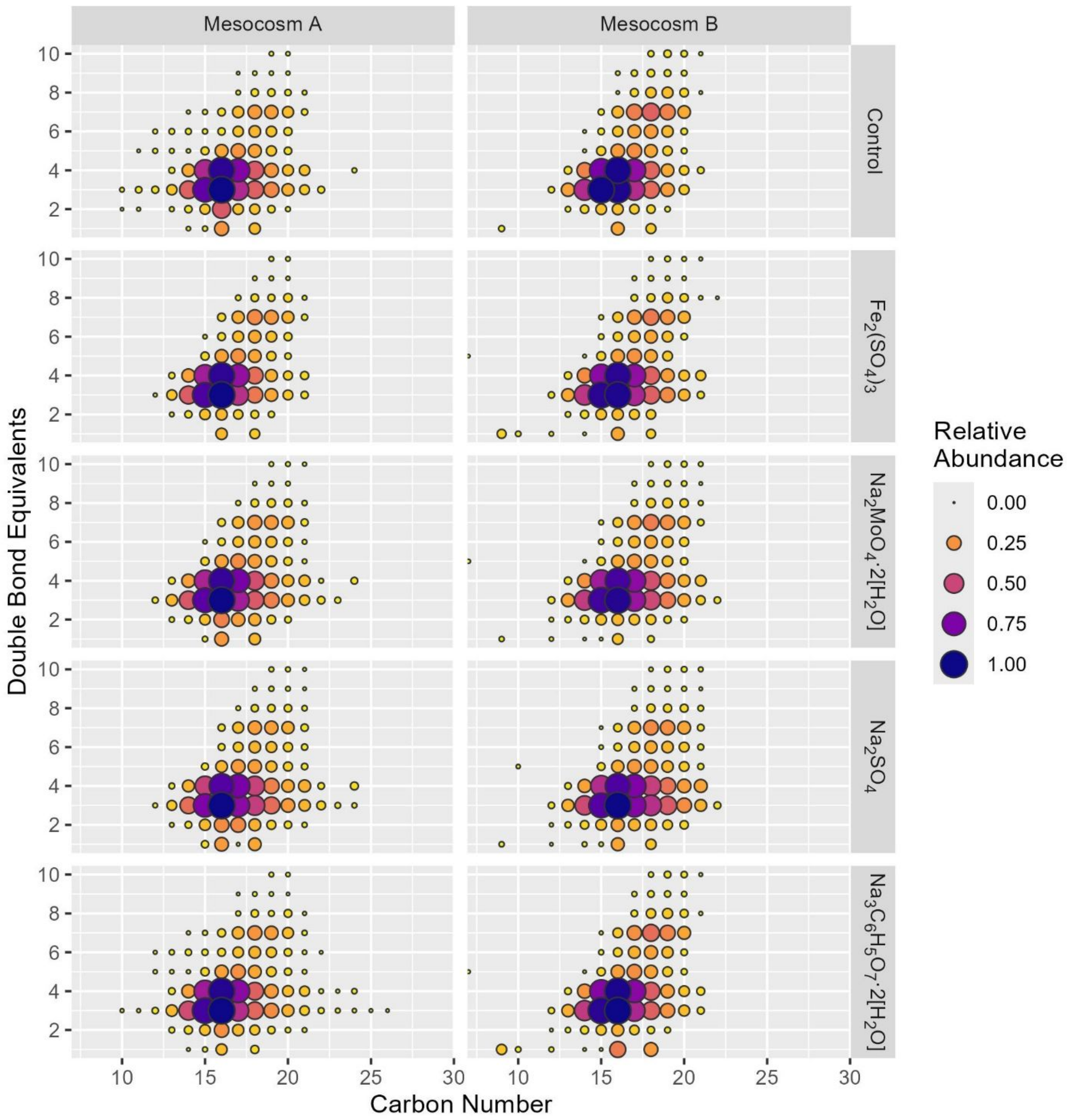

Figure S8 – Base peak-normalized abundance of O_2-NAFCs in mesocosms supplemented with naphthenic solvents in phase 3.

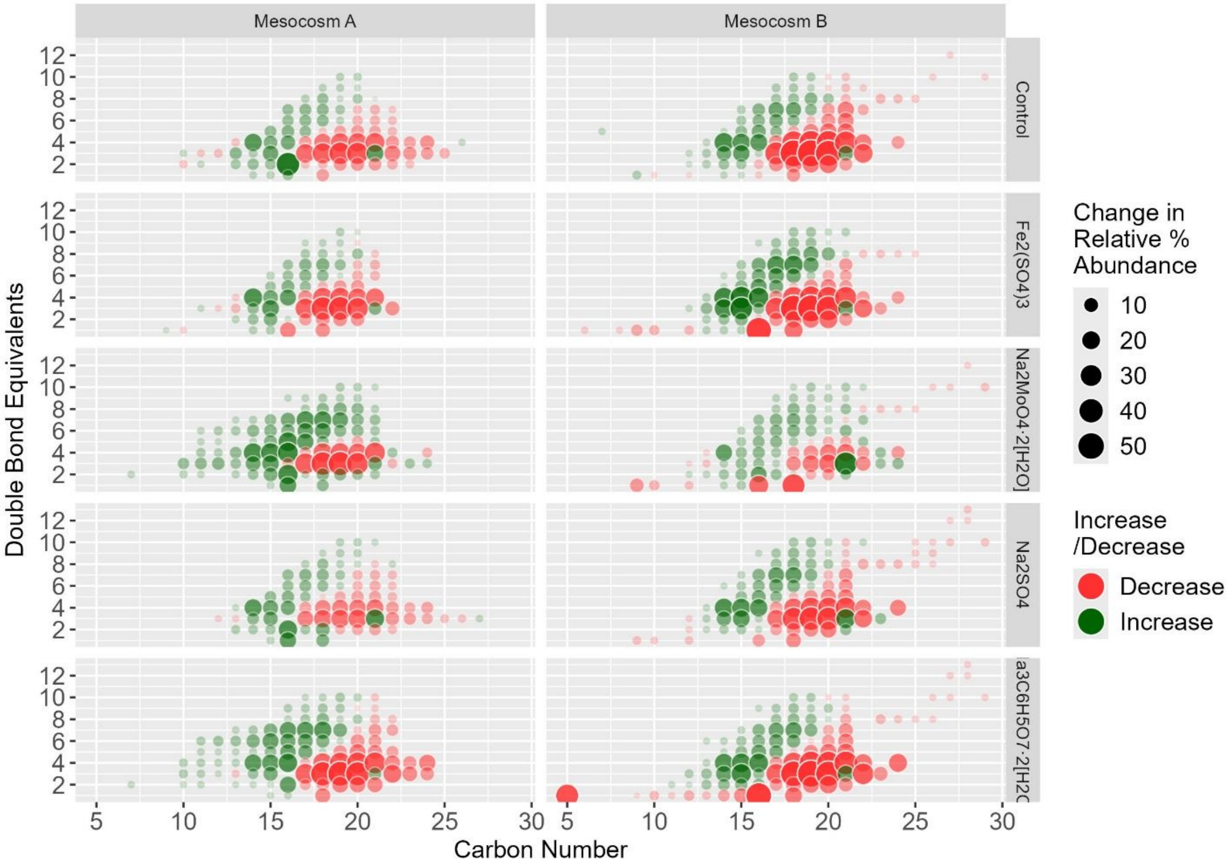

Figure S9 – A net-change carbon number versus double bond equivalents plot of naphthenic acids (i.e., O_2-NAFCs) detected by Orbitrap mass spectrometry from the beginning to end of the experimental period in mesocosms supplemented with paraffinic solvents.

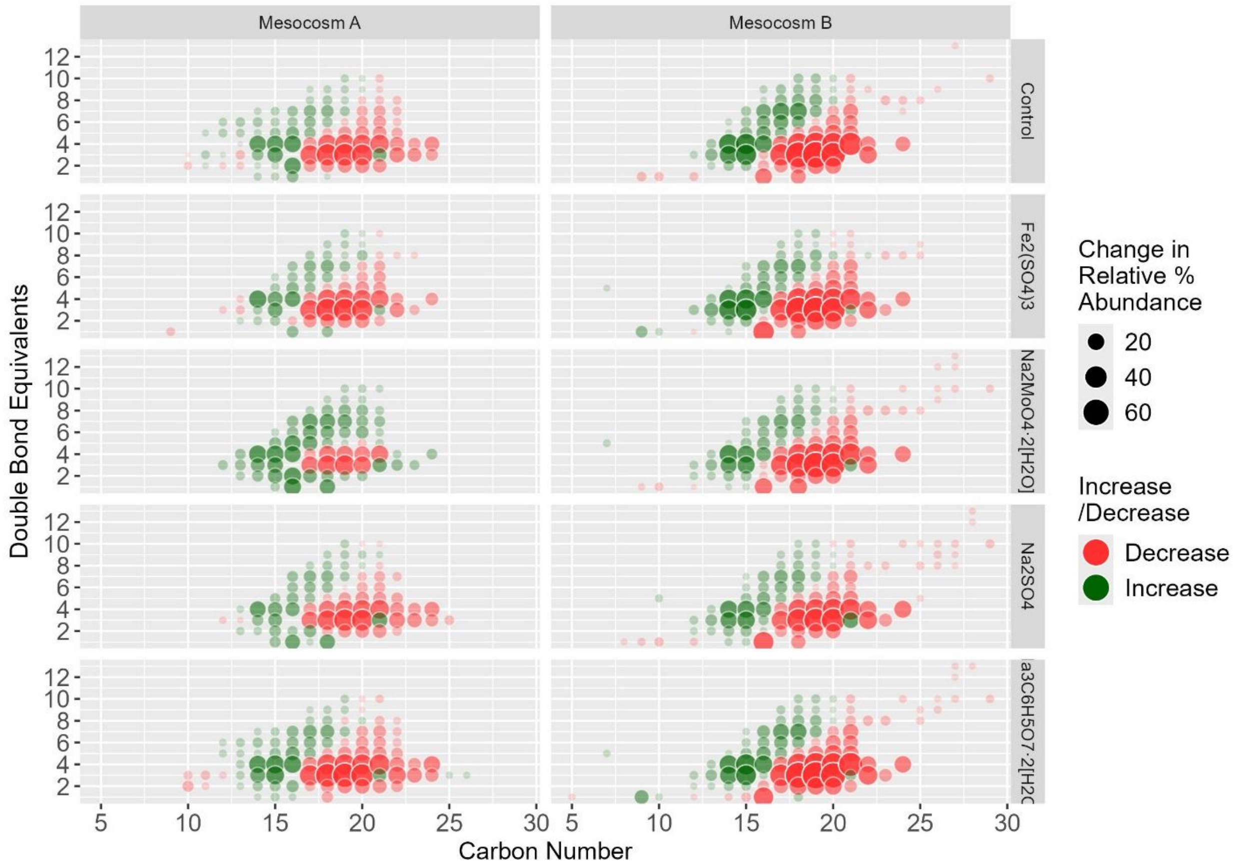

Figure S10 – A net-change carbon number versus double bond equivalents plot of naphthenic acids (i.e., O_2-NAFCs) detected by Orbitrap mass spectrometry from the beginning to end of the experimental period in mesocosms supplemented with naphthenic solvents.

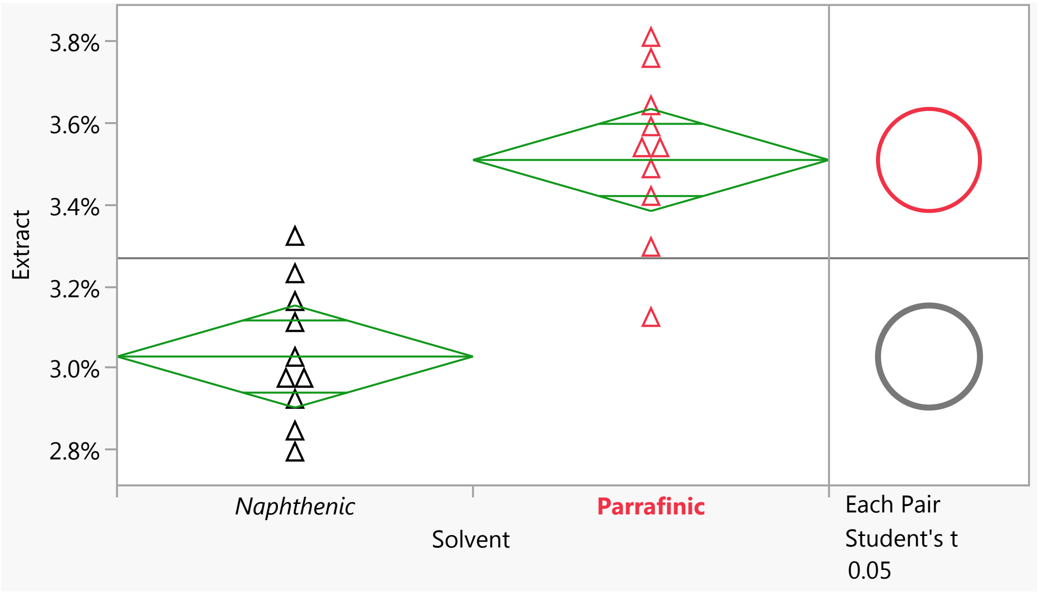

Figure S11 – One-way ANOVA analysis of Extract by the type of solvents presents in the Mesocosm B samples

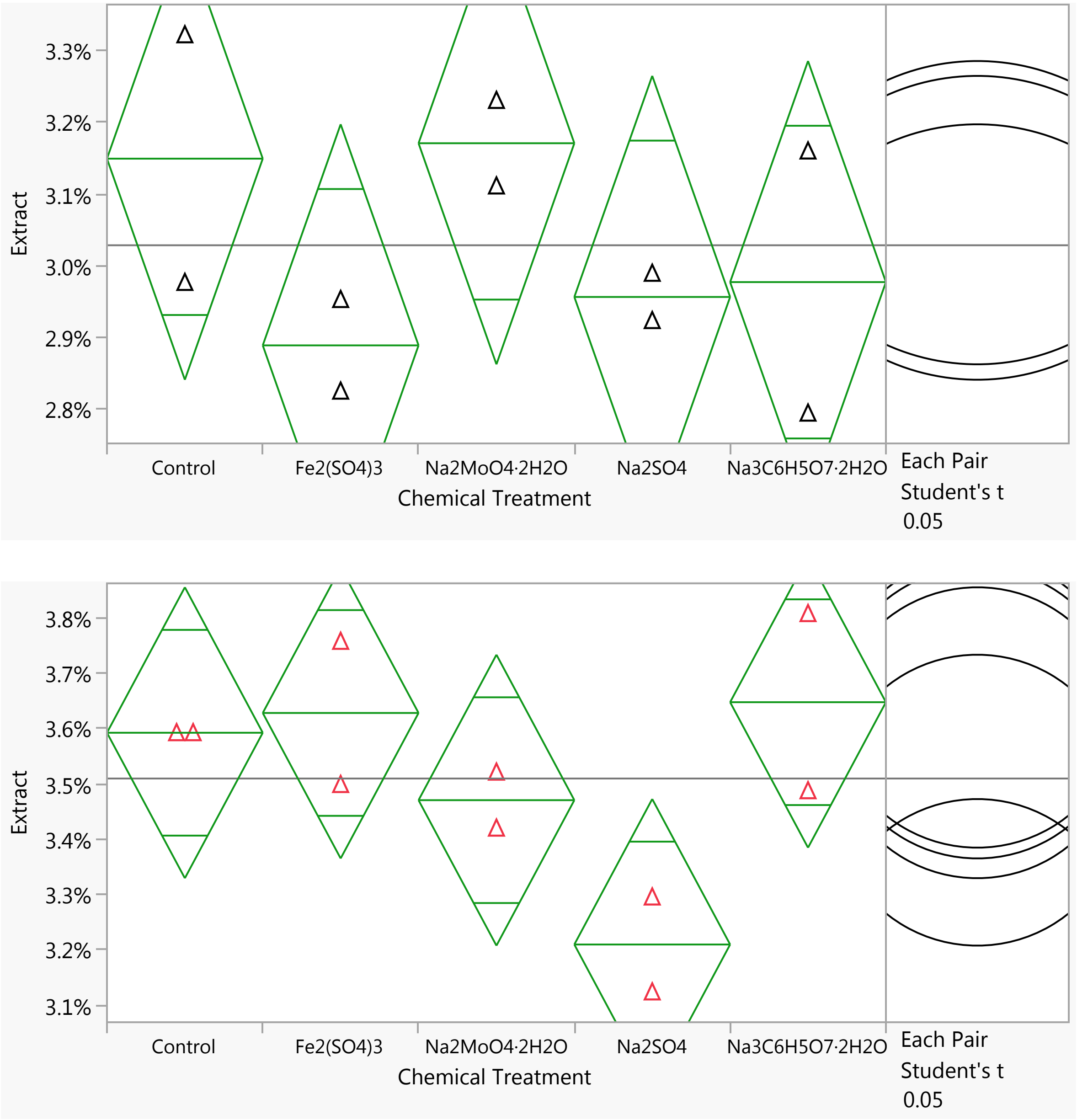

Figure S12 – One-way ANOVA Analysis of Extract by Chemical Treatment when naphthenic solvent (top) or paraffinic solvent (bottom) was present in the Mesocosm B tailing samples

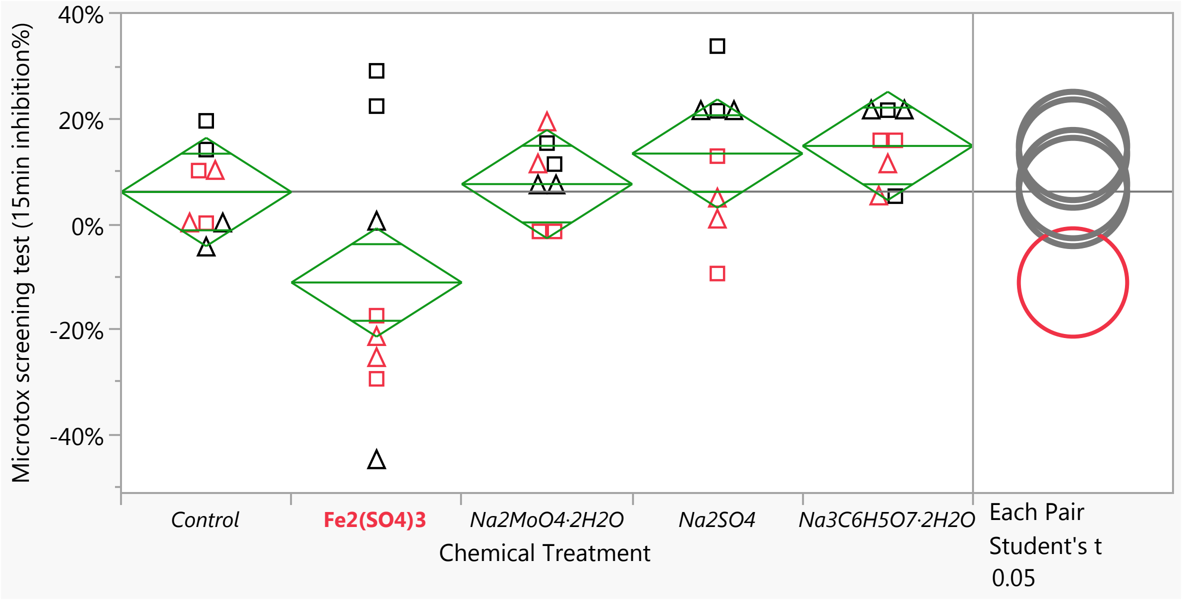

Figure S13 – One-way ANOVA analysis of Microtox screening test (inhibition% at 15min) results by chemical treatment. Mesocosm A samples are in square and Mesocosm B samples are in triangle; Paraffinic solvents in red and naphthenic solvents in black.

Table S1 – The positive/negative percentage changes of the NAFCs concentrations compared to each control

	Solvent	$Fe_2(SO_4)_3$	$Na_2MoO_4 \cdot 2H_2O$	Na_2SO_4	Sodium Citrate
**Mesocosm A**	Naphthenic	-54%	+22%	+3%	+31%
	Paraffinic	-35%	+55%	+5%	0%
**Mesocosm B**	Naphthenic	-31%	+30%	+55%	+3%
	Paraffinic	-18%	+72%	+57%	+15%
